# Development and Preclinical Characterization of [^18^F]H3-2406 and [^18^F]H3-2407 for Positron Emission Tomography Imaging of the Histamine Subtype‑3 Receptor

**DOI:** 10.1021/acs.jmedchem.4c02924

**Published:** 2025-07-16

**Authors:** Zhendong Song, Yinlong Li, Kenneth Dahl, Zhenkun Sun, Jiahui Chen, Xin Zhou, Yabiao Gao, Jian Rong, Chunyu Zhao, Katherine Yuan, Ahmad F. Chaudhary, Jimmy S. Patel, Thomas L. Collier, Chongzhao Ran, Kim S. Muehlfenzl, Achi Haider, Charles S. Elmore, Magnus Schou, Steven H. Liang

**Affiliations:** † Department of Radiology and Imaging Sciences, 1371 Emory University, 1364 Clifton Road, Atlanta, Georgia 30322, United States; ‡ PET Science Centre, Precision Medicine and Biosamples, Oncology R&D, AstraZeneca, Karolinska Institutet, Stockholm 17176, Sweden; § Department of Clinical Neuroscience, Centre for Psychiatry Research, Karolinska Institutet and Stockholm County Council, Stockholm 17176, Sweden; ∥ Department of Pharmacology and Chemical Biology, 12239 Emory University School of Medicine, Atlanta, Georgia 30322, United States; ⊥ Department of Radiation Oncology, Winship Cancer Institute of Emory University, Atlanta, Georgia 30322, United States; # Athinoula A. Martinos Center for Biomedical Imaging, Department of Radiology, 2348 Massachusetts General Hospital and Harvard Medical School, Boston, Massachusetts 02114, United States; ∇ Early Chemical Development, Pharmaceutical Sciences, R&D, AstraZeneca Pharmaceuticals, Gothenburg 43183, Sweden

## Abstract

The histamine subtype 3 receptor (H_3_R) is a G protein-coupled receptor involved in various central nervous system (CNS) disorders. We herein describe the identification and preclinical evaluation of two H_3_R antagonists: compounds **3** (H3-2406, *K*_i_ = 2.87 nM) and **4** (H3-2407, *K*_i_ = 3.15 nM) as potential PET radioligands. Both were radiolabeled with fluorine-18 using a copper-mediated method. Among them, [^18^F]**3** showed high radiochemical yield (32.4%), molar activity (103 GBq/μmol), and moderate brain uptake (SUV = 2.4), with regional distribution matching known H_3_R expression. [^18^F]**3** also exhibited favorable pharmacokinetics, high metabolic stability and negligible efflux by transporter proteins. However, relatively high cerebellar uptake, along with dedicated blocking studies, suggested off-target binding to the sigma-1 receptor, likely due to limited selectivity over sigma-1 (69-fold). These findings highlight [^18^F]**3** as a promising lead for H_3_R imaging, with future work aimed at improving its selectivity and minimizing off-target binding.

## Graphical abstract

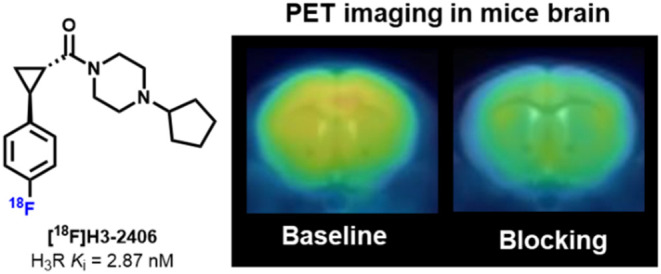

## Introduction

Histamine, a biogenic amine characterized by a simple nitrogen-containing heterocyclic structure, is involved in a multitude of physiological functions. In the central nervous system (CNS), histamine functions as a neurotransmitter with significant roles in the regulation of the sleep-wake cycle, learning, and memory via activation of four distinct histamine receptors: H_1_R, H_2_R, H_3_R, and H_4_R.^1–4^ Among these, H_3_R is recognized as a presynaptic inhibitory autoreceptor that modulates histamine synthesis and release,^5,6^ as well as a heteroreceptor regulating the release of neurotransmitters such as acetylcholine, serotonin, and dopamine.^7–9^ H_3_R is highly expressed in the human brain, including corpus striatum, cerebral cortex, hypothalamus, and hippocampus.^10,11^ Evidence from preclinical and clinical studies suggests H_3_R is implicated in several neurological and psychiatric conditions, including Alzheimer’s disease, Parkinson’s disease, sleep-wake disorders, neuropathic pain, and epilepsy.^12–16^ The therapeutic potential of H_3_R inverse agonists and antagonists has driven the development of several selective agents that have been evaluated in clinical trials, including AZD5213,^17^ GSK189254,^18^ JNJ-39220675,^19^ ABT-288,^20^ PF-03654746,^21^ and MK-7288 (Figure 1A).^22^

**Figure 1:**
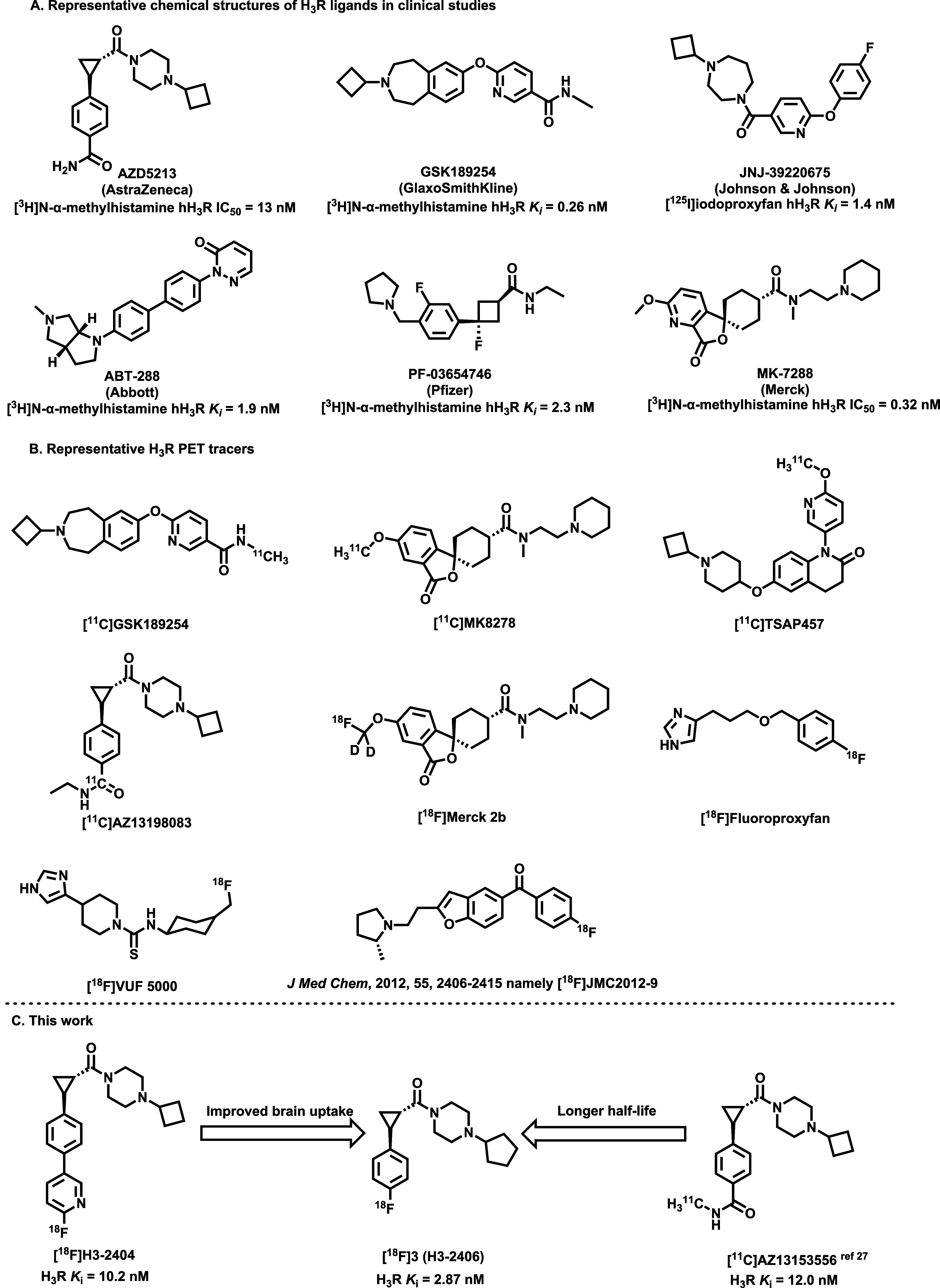
Representative H_3_R ligands and PET tracers. (A) The structures of H_3_R ligands in clinical studies; (B) reported H_3_R PET tracers; (C) this work, H_3_R binding affinity was measured by a radioligand binding assay using [^3^H]­AZ13582963.

Positron emission tomography (PET) is a valuable tool for understanding the distribution and function of H_3_R in both physiological and disease states and a suitable PET radioligand is essential for identifying potent and selective H_3_R drug candidates by enabling target occupancy studies. Several carbon-11 labeled compounds, such as [^11^C]­GSK189254,^23^ [^11^C]­MK-8278,^24^ [^11^C]­TASP457,^25,26^ [^11^C]­AZ13153556,^27^ and [^11^C]­AZ13198083,^28^ have been explored as H_3_R PET radioligands (Figure 1B,C), with [^11^C]­GSK189254, [^11^C]­MK-8278, and [^11^C]­TASP457 applied in human brain studies. However, the short half-life of carbon-11 (20.4 min) limits the broader clinical use of these radioligands. A few fluorine-18 (half-life: 109.8 min) labeled H_3_R radioligands have been reported, including [^18^F]­fluoroproxyfan,^29^ [^18^F]­VUF5000,^30^ [^18^F]­Merck 2b,^31^ and [^18^F]­JMC2012-9,^32^ however, none of these examples have successfully advanced to human studies may duo to significant limitations such as low brain uptake or poor metabolic stability. Therefore, developing fluorine-18 labeled PET radioligands with potent and selective H_3_R binding remains an unmet clinical need for effective H_3_R imaging.

We recently developed a fluorine-18 labeled H_3_R radioligand, [^18^F]­H3-2404, inspired by the structure of AZD5213 (Figure 1C).^33^ However, this probe exhibited limited and nonspecific brain uptake in rodents, impeding further development. We hypothesized that the poor brain uptake may stem from the pyridine motif, which increases polarity and hydrogen bonding capacity, thereby potentially reducing blood-brain barrier permeability. Further, pyridinyl nitrogen may enhance recognition by efflux transporters such as P-glycoprotein, further limiting brain penetration. Of note, the introduction of this motif can negatively impact binding affinity to H_3_R. In the present study, we modified the scaffold of [^18^F]­H3-2404 by removing the pyridine group, while maintaining amenability for fluorine-18 labeling. The optimization of cyclopropyl formamide with different nonaromatic nitrogen heterocyclic rings resulted in a series of novel H_3_R candidates. Pharmacological evaluation identified compounds **3** (H3-2406, *K*_i_ = 2.87 nM) and **4** (H3-2407, *K*_i_ = 3.15 nM) with high binding affinity for H_3_R and CNS target selectivity (over 300-fold against most CNS targets). Fluorine-18 labeling of both two compounds gave [^18^F]**3** ([^18^F]­H3-2406) and [^18^F]**4** ([^18^F]­H3-2407), which were evaluated by autoradiography, PET imaging, whole-body *ex vivo* biodistribution, and radiometabolite analysis. This study provides a comprehensive preclinical assessment of both radioligands, positioning [^18^F]**3** as a lead candidate for PET imaging in the brain.

## Results and Discussion

### Chemical Synthesis and Pharmacology

To develop H_3_R antagonists with enhanced potency and selectivity, we designed and synthesized a series of analog compounds **1**–**9** derived from the structure of H3-2404. The modifications focused on the cyclopropyl formamide and phenyl groups to improve brain uptake. As depicted in Scheme 1, compounds **1**–**7** were prepared in yields ranging from 40.6–75.4% through the condensation reactions of commercially available intermediate **10** with different amine agents **11a**–**11g**. Compound **8** was obtained in an overall yield of 12.9% via the condensation reactions of intermediate **10** with reagent **12**, followed by the removal of the protecting *N*-Boc group and subsequent reductive amination reaction with **15**.

**Scheme 1:**
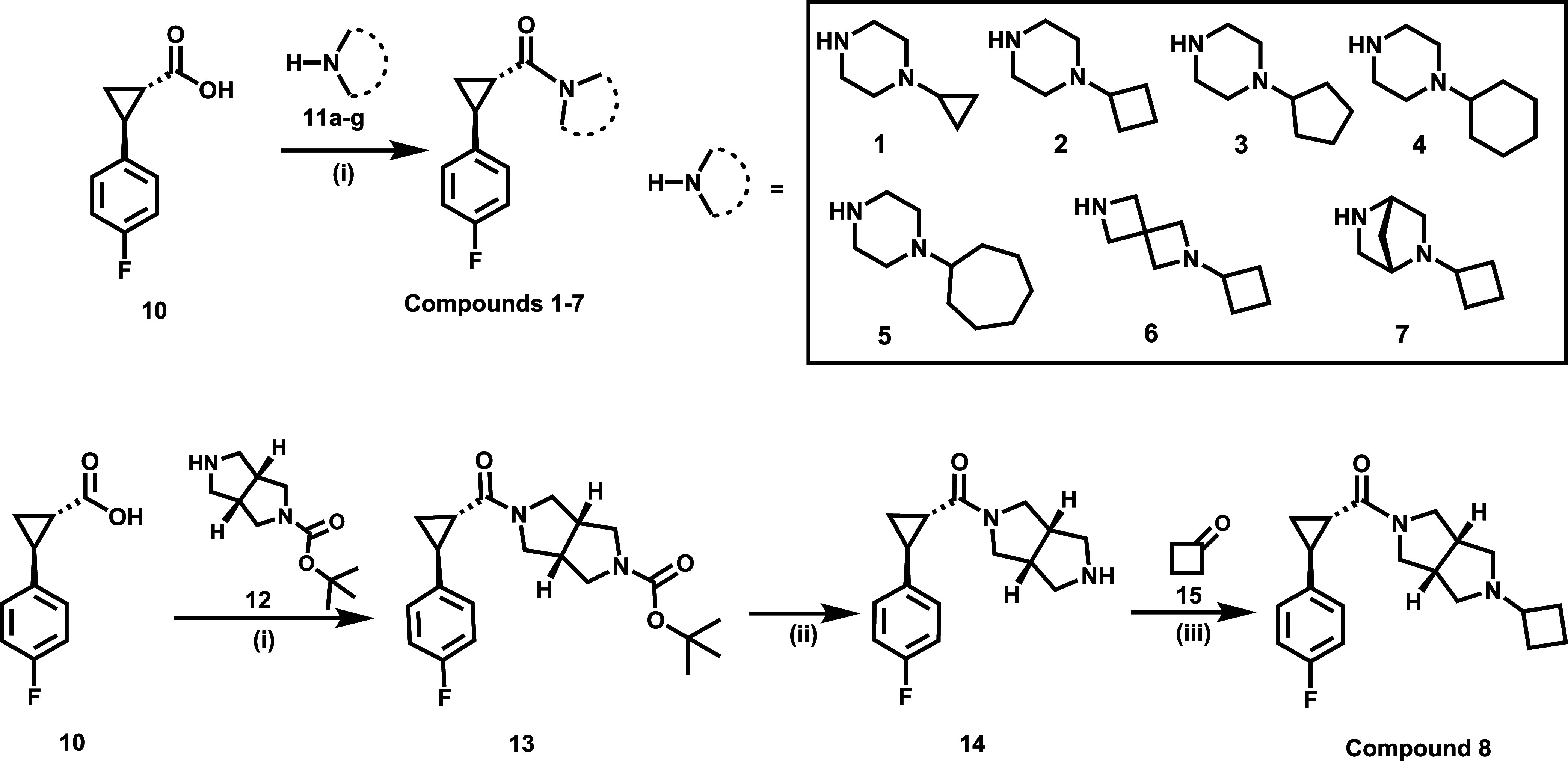
Synthesis of H_3_R Candidates **1**–**8** ^a^

a Reagents and conditions: (i) DIEA, HATU, DMF, 25 °C, N_2_, 4–12 h, 40.6–75.4% yields (ii) TFA, DCM, 25 °C, N_2_, 12 h; (iii) TEA, AcOH, MeOH, 25 °C, 1 h, then NaBH_3_CN, 0 to 25 °C, N_2_, 11 h, 12.9% total yield.

The synthetic route of compound **9** is outlined in Scheme 2. Starting from commercially available compound **16**, condensation with amine **11b** yielded intermediate **17**. Subsequently, key intermediate **18** was obtained through a carbonylative cross coupling reaction under a carbon monoxide atmosphere. After hydrolysis, intermediate **19** was then coupled with azetidine **20** in the presence of hexafluorophosphate azabenzotriazole tetramethyl uronium (HATU) to afford compound **9** in 41.8% yield.

**Scheme 2:**
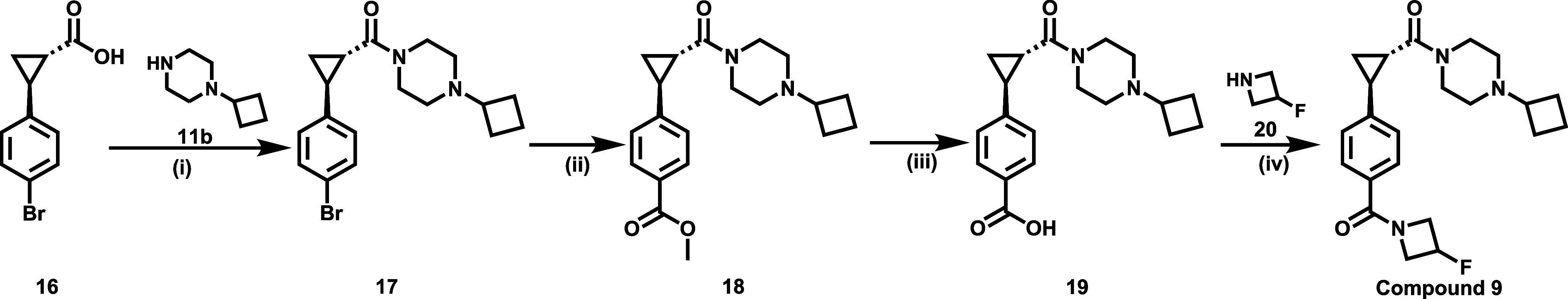
Synthesis of H_3_R Candidate **9** ^a^

a Reagents and conditions: (i) DIEA, HATU, DMF, 25 °C, N_2_, 18 h, 97.7% yield; (ii) TEA, CO (50 psi), Pd­(dppf)­Cl_2_, MeOH, 80 °C, 60 h, 75.4% yield; (iii) LiOH·H_2_O,THF/MeOH (2:1), 25 °C, N_2_, 5 h, 60.7% yield; (iv) DIEA, HATU, DMF, 25 °C, N_2_, 3 h, 41.8% yield.

To evaluate the binding affinities of compounds **1**–**9**, competitive radioligand binding assays were conducted using [^3^H]­AZ13582963 [4-(1*S*,2*S*)-2-(4-cyclobutylpiperazine-1-carbonyl)­cyclopropyl)-*N*-methyl-[2-^3^ *H*]-benzamide]. As shown in Table 1, compound **1**, featuring a cyclopropyl-substituted piperazine, exhibited moderate H_3_R affinity (*K*_i_ = 71.3 nM). Substitution of the cyclopropyl group on the piperazine with cyclobutyl (compound **2**), cyclopentyl (compound **3**), and cyclohexyl (compound **4**) groups significantly improved H_3_R potency with the *K*_i_ values of 2.46 nM, 2.87 nM, and 3.15 nM, respectively. Conversely, compound **5**, which incorporated a bulkier cycloheptyl group on the piperazine ring, lost inhibitory activity (*K*_i_ > 1000 nM). These results indicated that *C*4–*C*6 cycloalkanes side chains were beneficial for retaining the H_3_R inhibitory potency. Further structural modifications were tested by replacing the piperazine ring with 2,6-diazaspiro[3.3]­heptane, 2,5-diazabicyclo[2.2.1]­heptane, and 2-octahydrocyclopenta­[*c*]­pyrrole (compounds **6**–**8**), resulting in low affinities (*K*_i_ > 1000 nM). Additionally, the substitution of the 6-fluoropyridine group of H3-2404 with a 3-fluoroazetidine group (compound **9**, *K*_i_ = 98.3 nM) had a minimal impact on potency. Based on the structure–activity relationships (SAR), compounds **2**, **3**, and **4** emerged as promising candidates for H_3_R PET imaging ligands.^34,35^ The lipophilicity of **2**, **3**, and **4** were measured by the “shake-flask” method, yielding log *D* values of 2.76, 2.82, and 3.35, respectively.^36^ Further, ACD Percepta predictions indicated these compounds are likely to cross the blood-brain barrier (BBB), as indicated by a log BB > −1 and a multiparameter optimization (MPO) score >4.0.^37^ High selectivity toward H_3_R over other CNS targets is essential for effective H_3_R PET imaging. Therefore, off-target screenings of compounds **2**, **3**, and **4** were conducted against various CNS receptors, G protein-coupled receptors (GPCRs), transporters, and ion channels at 10 μM (Figure S1). Compound **2** demonstrated high selectivity, except for the sigma-1 receptor, which was inhibited by 98.1% at 10 μM. Similarly, compound **3** demonstrated inhibitory effects on sigma-1 (93.2%) and 5-hydroxytryptamine receptor 2A (5-HT_2A_, 64.5%), while compound **4** showed inhibition of 94.4% for sigma-1, 93.6% for sigma-2, 59.0% for 5-HT_2A_, and 80.3% for 5-HT_2C_. Further binding assays of sigma-1 revealed the *K*_i_ values of compounds **2**, **3**, and **4** to be 1.61 nM (0.65-fold selectivity of H_3_R), 199 nM (69-fold selectivity of H_3_R), and 1282 nM (407-fold selectivity of H_3_R), respectively (Figure S2A). In subsequent 5-HT_2C_ binding assays, compounds **3** and **4** showed *K*_i_ values >1000 nM, indicating a high selectivity of these two compounds over 5-HT_2C_ (Figure S2B). Based on these findings, compound **2** was identified as a dual antagonist for H_3_R and sigma-1 receptors. Sigma-1 receptors, crucial for ion channel function and associated with learning, memory, and pain,^38,39^ have shown synergy with H_3_R antagonists, including PF-03654746 and ABT-239, potentially enhancing *in vivo* efficacy.^5^ Compounds **3** and **4**, demonstrating suitable pharmacological profiles and target selectivity, were selected for radiolabeling and further evaluation as H_3_R PET imaging agents.

**Table 1:**
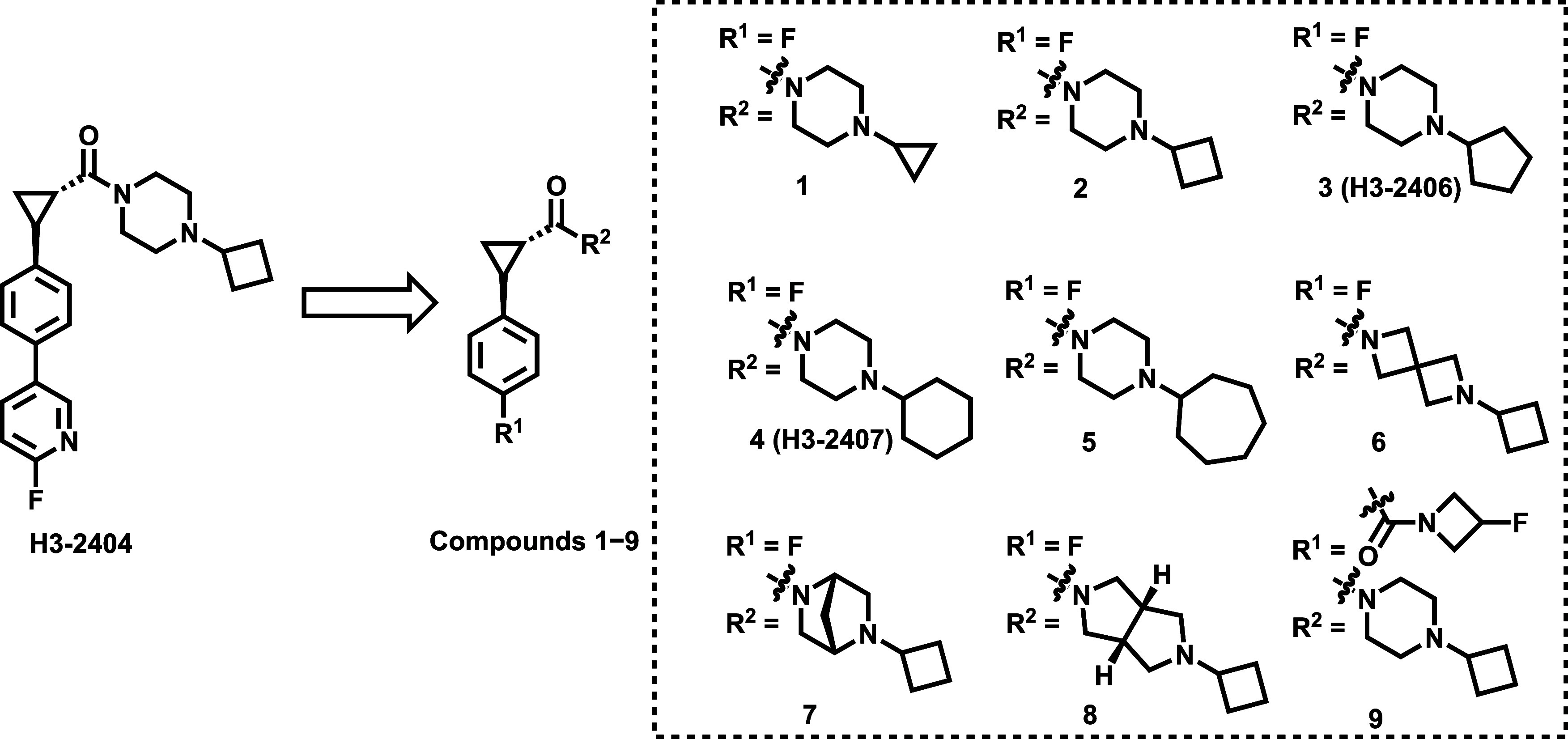
PET-Specific SAR Optimization, Pharmacology and Physiochemical Properties of Compounds **1**–**9**

compounds	H_3_R *K*_i_ (nM)	H_3_R IC_50_ (nM)	MW^a^	c log *P* ^a^	tPSA^a^	MPO score^b^	log BB^b^	log *D*
1	71.3	134	288	2.41	23.6	5.2	0.28	
2	2.46	4.80	302	2.74	23.6	4.8	0.37	2.76
3 (H3-2406)	2.87	5.60	316	3.30	23.6	4.7	0.28	2.82
4 (H3-2407)	3.15	6.15	330	3.86	23.6	4.5	0.68	3.35
5	>1000	>1000	344	4.41	23.6	3.8	0.93	
6	>1000	>1000	314	2.64	23.6	4.6	0.62	
7	>1000	>1000	314	3.09	23.6	4.3	0.17	
8	>1000	>1000	328	2.30	23.6	4.2	0.59	
9	98.3	198	385	1.43	43.9	5.8	0.04	
AZD13153556	12.0	23.5	341	1.31	52.7			

a Values were calculated with ChemDraw 22.2 software.

b Values were predicted with ACD/laboratories.

### Radiochemistry

To synthesize the ^18^F-labeled analogs of compounds **3** and **4**, precursors **22** and **24** with corresponding boronate esters on the phenyl ring were prepared via two-step reactions (Scheme 3). Initially, commercially available **16** was reacted with substituted piperazines **11c** and **11d** to afford intermediates **21** and **23**. The subsequent Pd-catalyzed Suzuki coupling reaction generated **22** and **24** as precursors with 25.6 and 26.7% yields, respectively. Following established methods,^40–42^ the radiolabeling of **22** and **24** was achieved by treating a mixture of precursor (1.5 mg), [^18^F]­Et_4_NF, Cu­(OTf)_2_Py_4_ (8 mg) in DMA/*n*-BuOH (300 μL, 2:1, v/v) at 120 °C for 15 min. Radioligands [^18^F]**3** and [^18^F]**4** were obtained in high nondecay corrected radiochemical yields (RCY) of 32.4 and 22.6%, respectively. Both radioligands were obtained by HPLC purification with high radiochemical purities (>99%) and good to excellent molar activities (Figure S4, 103 GBq/μmol of [^18^F]**3** and 30 GBq/μmol of **[** ^**18**^ **F]­4**), enabling their subsequent *in vitro* and *in vivo* evaluation.

**Scheme 3:**
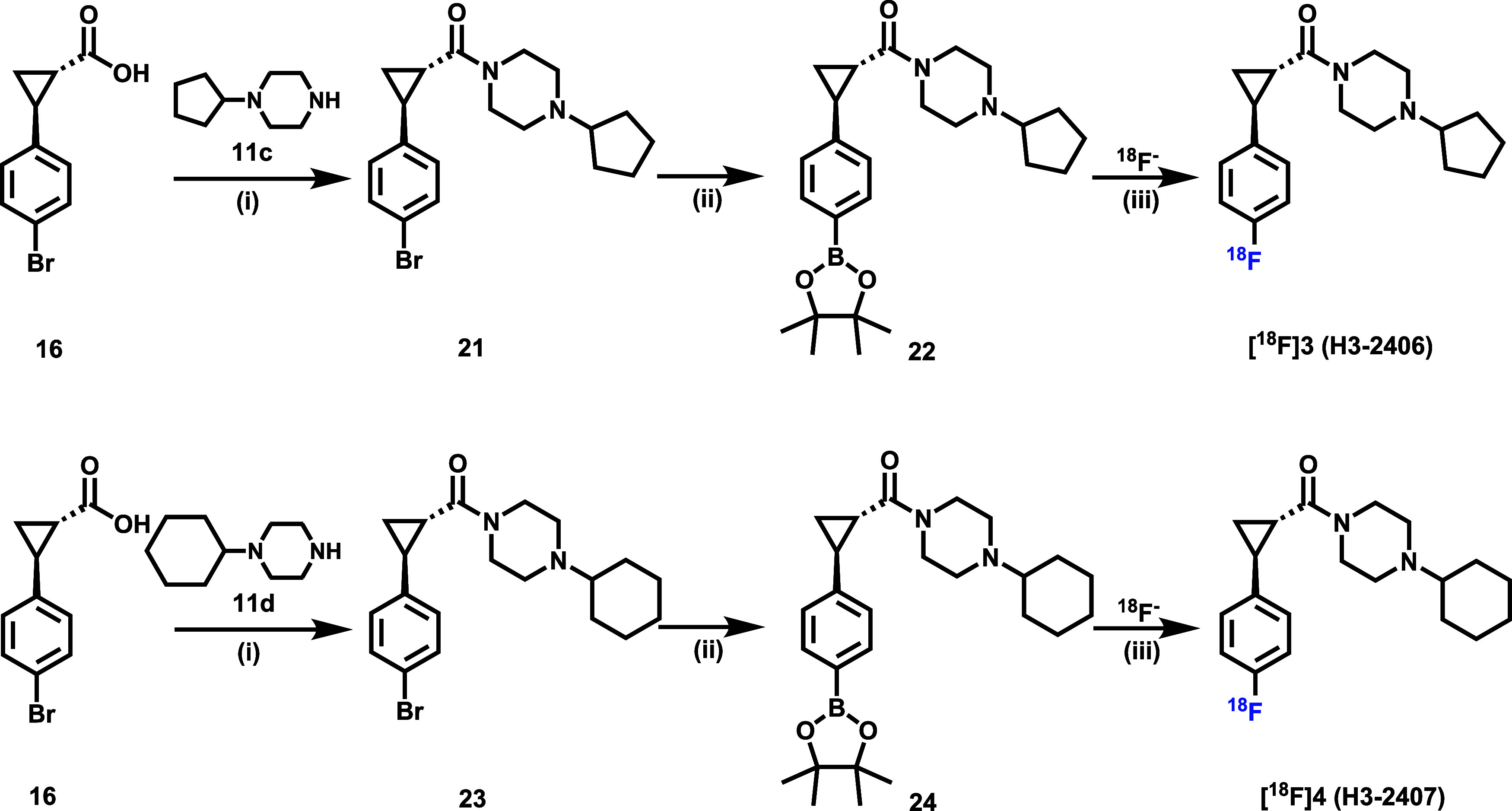
Synthesis of Precursor **22**, **24** and Radiosynthesis of [^18^F]**3** and [^18^F]**4** ^a^

a Conditions: (i) DIEA, T3P, DCM, 25 °C, 2 h; (ii) BPD, Pd­(dppf)­Cl_2_, AcOK, dioxane, 100 °C, 3 h, 25.6–26.7% yields of two steps; (iii) TEAB, Cu­(OTf)_2_Py_4_, [^18^F]­HF, DMA/*n*-BuOH (2:1), N_2_, 120 °C, 15 min, 32.4% nondecay-corrected RCY for [^18^F]**3**, 22.6% nondecay-corrected RCY for [^18^F]**4**.

### *In Vitro* Autoradiography

To validate the target specificity and selectivity, *in vitro* autoradiography experiments were conducted using [^18^F]**3** and [^18^F]**4** on rat brain sections. As depicted in Figure 2A,C, incubation with [^18^F]**3** under baseline conditions revealed heterogeneous regional distributions, characterized by relatively high signals in the cortex, striatum, hippocampus, hypothalamus, largely consistent with the distribution of H_3_R in the rat brain.^32,43^ Blocking studies with GSK189254 (10 μM), an H_3_R antagonist, resulted in substantial reductions in radioactivity across various brain regions (range 43.6–86.0%), indicating that [^18^F]**3** preferentially targets H_3_R. Furthermore, pretreatment with unlabeled compound **3** (10 μM) led to a signal reduction of over 90% in the brain. Notably, self-blocking with unlabeled **3** resulted in greater signal reduction than GSK189254. This discrepancy is likely attributable to additional off-target binding of [^18^F]**3**, which is further supported by high uptake in the cerebellum – a region with low H_3_R expression. Similarly, [^18^F]**4** showed a heterogeneous distribution in the rat brain with relatively high signals in the cortex, striatum, hypothalamus, and hippocampus, with notably elevated radioactivity in the cerebellum during the baseline study (Figure 2B,D). The high uptake in the cerebellum may be attributed to nonspecific binding to unidentified CNS targets. Subsequent blocking of [^18^F]**4** with GSK189254 (38.7–68.2%) and unlabeled **4** (66.6–78.8%) effectively reduced brain uptake. Overall, these findings indicate that while [^18^F]**3** and [^18^F]**4** exhibit significant binding to H_3_R, some off-target binding was observed by *in vitro* autoradiography experiments. Nonetheless, the results support further *in vivo* PET evaluation to better understand their binding profiles and evaluate their potential for H_3_R imaging – particularly in regions with low sigma-1 abundance.

**Figure 2:**
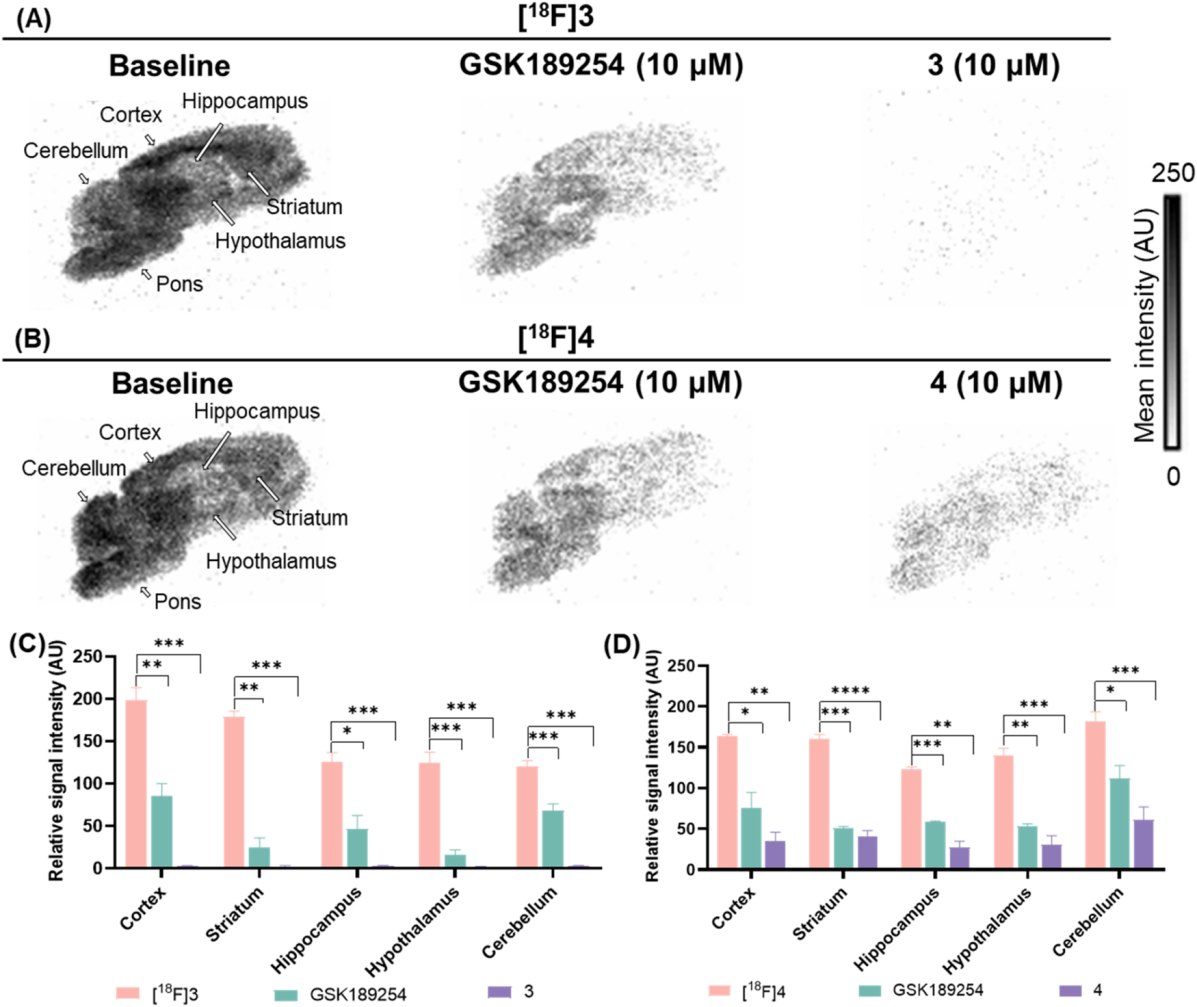
*In vitro* autoradiography of [^18^F]**3** and [^18^F]**4** in rat brain sections. (A) Representative images for baseline, GSK189254-blocking (10 μM), and unlabeled **3**-blocking (10 μM) studies with [^18^F]**3**. (B) Representative images for baseline, GSK189254-blocking (10 μM), and unlabeled 4-blocking (10 μM) studies with [^18^F]**4**. (C) Quantification of autoradiography studies with [^18^F]**3**. (D) Quantification of autoradiography studies with [^18^F]**4**. All data were referred to as mean ± SD, *n* = 3. *P ≤ 0.05, **P ≤ 0.01, ***P ≤ 0.001, ****P ≤ 0.0001.

### PET Imaging Studies of [^18^F]**3** and [^18^F]**4**

To elucidate the *in vivo* brain uptake of [^18^F]**3** and [^18^F]**4**, dynamic PET imaging studies were conducted in CD1 mice (an outbred strain of immunocompetent mice commonly used for pharmacokinetic and biodistribution studies) for 60 min. The results, which include summed PET images (0–60 min), whole-brain time-activity curves (TACs), and area under the curve (AUC) for both radioligands, are depicted in Figures 3 and 4. At baseline, [^18^F]**3** rapidly crossed the BBB following intravenous injection, reaching peak whole-brain uptake with a standardized uptake value (SUV) value of 2.4 at 2.3 min. This was followed by a gradual washout and reached a SUV of 1.87 by 60 min. To evaluate the binding characteristics of [^18^F]**3**, elective H_3_R antagonists with distinct structural scaffolds, GSK189254, enerisant, and cipralisant, were used as blocking agents. Each of these antagonists has been previously validated for H_3_R specificity, and their selectivity profiles have been documented in prior studies.^5,44,45^ In mice pretreated with GSK189254 (3 mg/kg), brain radioactivity peaked at 1.98 SUV at 1.9 min, subsequently declining to 1.27 SUV by 60 min, indicating a signal reduction of 32.5% based on AUC. Preblocking with other selective H_3_R antagonists, enerisant (3 mg/kg) and cipralisant (3 mg/kg), resulted in comparable blocking effects, with AUC reductions of 35.6% and 38.4%, respectively (Figure 3B,C), substantiating the conclusion that [^18^F]**3** binds to H_3_R *in vivo*. Quantification of specific tracer binding was performed using volume of distribution (*V*_T_) values, derived from compartmental modeling and an image-derived input function (IDIF). This approach provided an estimation of *V*_T_ values and demonstrated a significant reduction in H_3_R-rich brain regions such as the cortex, striatum, thalamus, and hippocampus under blocking conditions with GSK189254, enerisant, and cipralisant (Table S1). While these findings support the utility of [^18^F]**3** for *in vivo* studies, we acknowledge the inherent limitations of IDIF, including the lack of metabolite correction and potential inaccuracies in capturing early phase kinetics. Notably, previous studies have demonstrated that graphical Logan analysis provides more reliable *V*_T_ estimates than compartmental modeling when an IDIF is employed.^46,47^ Given these considerations, Logan analysis was selected as the preferred approach in this study to mitigate potential biases associated with IDIF-based quantification.^48^ However, we observed that blocking with selective H_3_R antagonists resulted in an incomplete reduction in radioactivity signal, indicating potential off-target binding of [^18^F]**3**. In particular, compound **3** demonstrated only 69-fold selectivity over sigma-1 receptors, which could have been insufficient in brain areas with relatively high sigma-1 abundance. To further investigate this, we conducted additional *in vivo* PET imaging studies using NE-100, a selective sigma-1 receptor antagonist, which led to a partial reduction in brain uptake. Furthermore, preinjection of ABT-239, a dual H_3_R and sigma-1 receptor antagonist, resulted in near-complete signal blockade, consistent with the radioactivity reduction observed in self-blocking studies using unlabeled compound **3** (Figure S3). These results strongly suggest that, despite the high H_3_R binding of [^18^F]**3**, a significant component of signal retention in the brain is due to off-target interaction with the sigma-1 receptor.

**Figure 3:**
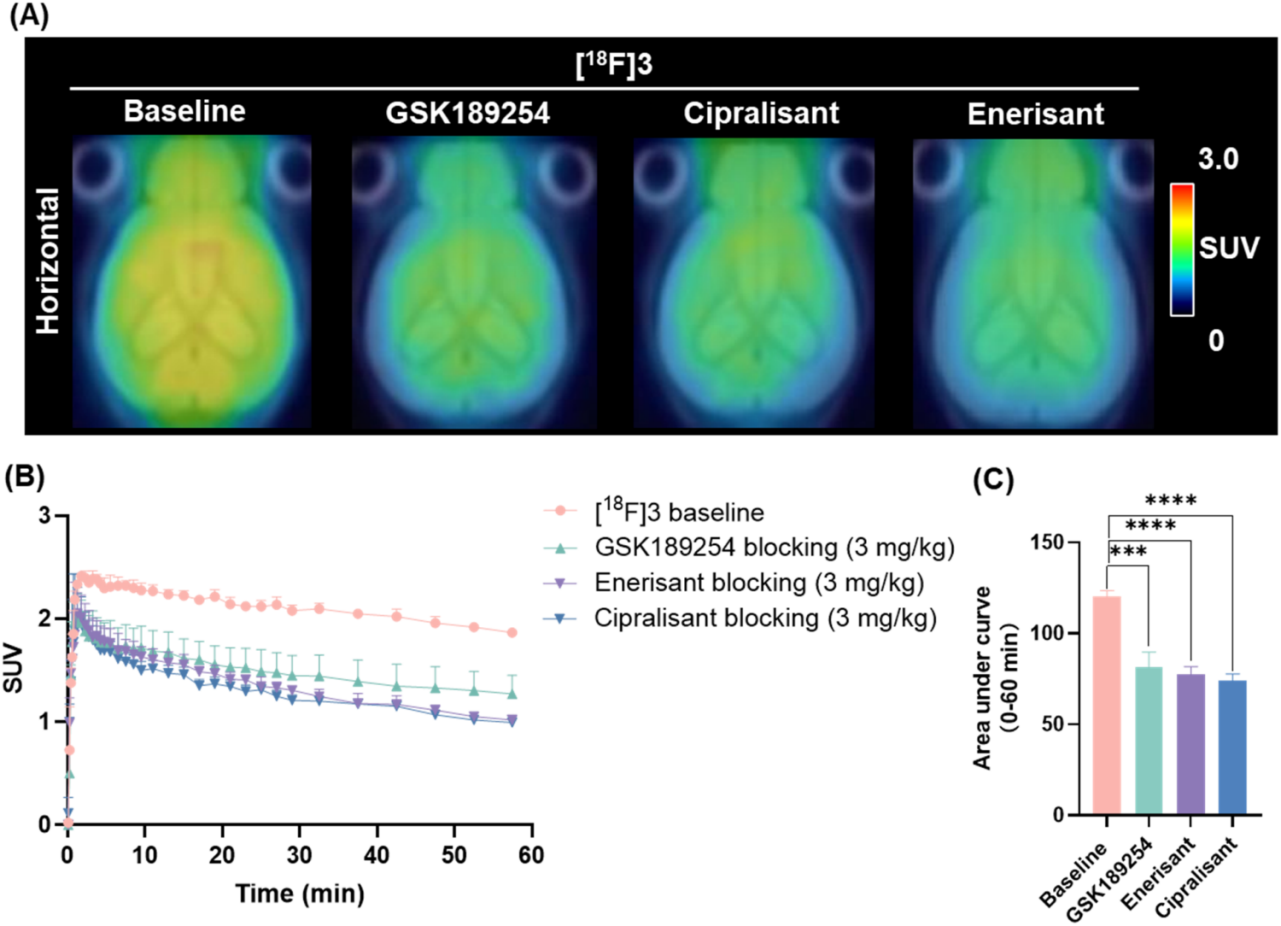
Mice PET imaging studies (0–60 min) of [^18^F]**3**. (A) Representative summed PET images of brain uptake under baseline and GSK189254-blocking (3 mg/kg), enerisant-blocking (3 mg/kg), and cipralisant-blocking (3 mg/kg) conditions; (B) TACs of [^18^F]**3** in the whole-brain; (C) Area under curve of [^18^F]**3** in the whole-brain; All data were referred to as mean ± SD, *n* = 4, asterisks indicate the statistical significance, ***P ≤ 0.001, ****P ≤ 0.0001.

**Figure 4:**
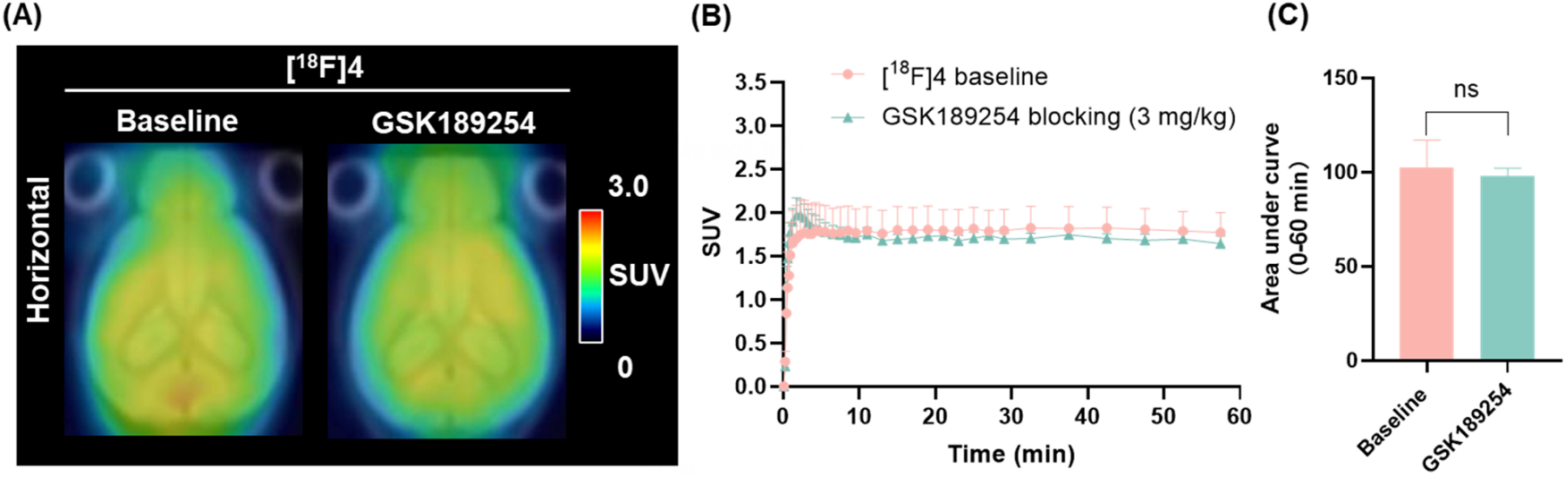
Mice PET imaging studies (0–60 min) of [^18^F]**4**. (A) Representative summed PET images of brain uptake under baseline and GSK189254-blocking (3 mg/kg) conditions; (B) TACs of [^18^F]**4** in the whole-brain; (C) Area under curve of [^18^F]**4** in the whole-brain; All data were referred to as mean ± SD, *n* = 4, ns, not significant.

To determine whether [^18^F]**3** is actively effluxed by BBB transporters such as P-glycoprotein (P-gp) and breast cancer resistance protein (BCRP), we conducted additional PET scans in mice pretreated with elacridar (3 mg/kg, a dual P-gp/BCRP inhibitor) 20 min prior to radioligand injection. While brain uptake of [^18^F]**3** did not significantly change overall, a trend toward higher uptake was noted, suggesting a potential minor contribution of efflux transporters. However, the effect was not significant, indicating that further optimization targeting efflux transporter liabilities would likely not provide substantial additional benefit (Figure S4).

For [^18^F]**4**, whole-brain radioactivity peaked at 1.83 SUV at 4.3 min; however, only a 3.3% reduction (1.77 SUV) in uptake was observed after 60 min dynamic scan. Blocking studies with GSK189254 (3 mg/kg) did not yield a significant reduction of whole-brain radioactivity for [^18^F]**4** (Figure 4). This observation may be, at least in part, attributed to physicochemical properties, as reflected by the LogD values of [^18^F]**4** (3.35) and [^18^F]**3** (2.82). Given that [^18^F]**4** contains a more lipophilic cyclohexyl moiety, it is likely that its increased nonspecific uptake in brain tissue, particularly white matter, contributed to the attenuation of the blocking effect. These findings suggest that the performance differences between [^18^F]**3** and [^18^F]**4** are likely driven by nonspecific binding.

### Whole-Body Biodistribution Studies of [^18^F]**3**

To further investigate the whole-body distribution of [^18^F]**3**, *ex vivo* biodistribution experiments were conducted in CD1 mice at 5-, 15-, 30-, and 60 min postadministration. A rapid and substantial brain uptake of 10.9 %ID/g was observed at 5 min, followed by a gradual washout over 60 min (Figure 5). Initially, high levels of radioactivity were detected in the heart, lung, and kidney, with subsequent clearance observed in these organs over time. At later time points, elevated uptake was noted in the liver and intestine, suggesting a possible hepatobiliary elimination pathway for [^18^F]**3**. Minimal radioactivity was detected in the blood and bone at all time points throughout the study. Specifically, radioactivity in bone remained approximately at 2 %ID/g, corresponding to less than 1 SUV, and did not show a significant increase over time. These findings indicate that our tracer is relatively stable against *in vivo* defluorination.

**Figure 5:**
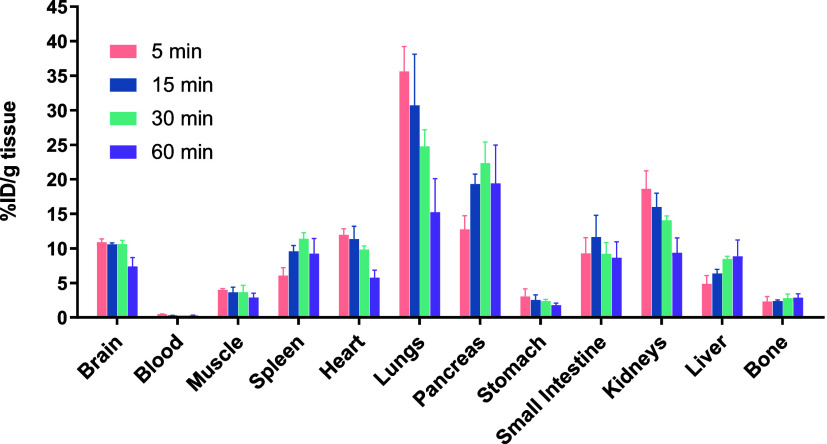
Whole-body *ex vivo* biodistribution studies of [^18^F]**3** in CD1 mice. All data are presented as mean ± SD, *n* = 4.

### Stability of [^18^F]**3** *In Vitro* and *Ex Vivo*

To assess the stability of [^18^F]**3**, *in vitro* radioactivity analyses were conducted following 30-, 60- and 90 min coincubation of the radioligand in the serum and liver microsomes from mice, nonhuman primates (NHPs), and humans (Figure 6). [^18^F]**3** demonstrated high stability in the serum across all species at different time points, with over 95% of the parent compound remaining intact. Additionally, the parent fractions in liver microsomes were found to be 78%, 85%, and 95% for mice, NHPs and humans at 90 min, respectively, indicating reasonable *in vitro* stability.

**Figure 6:**
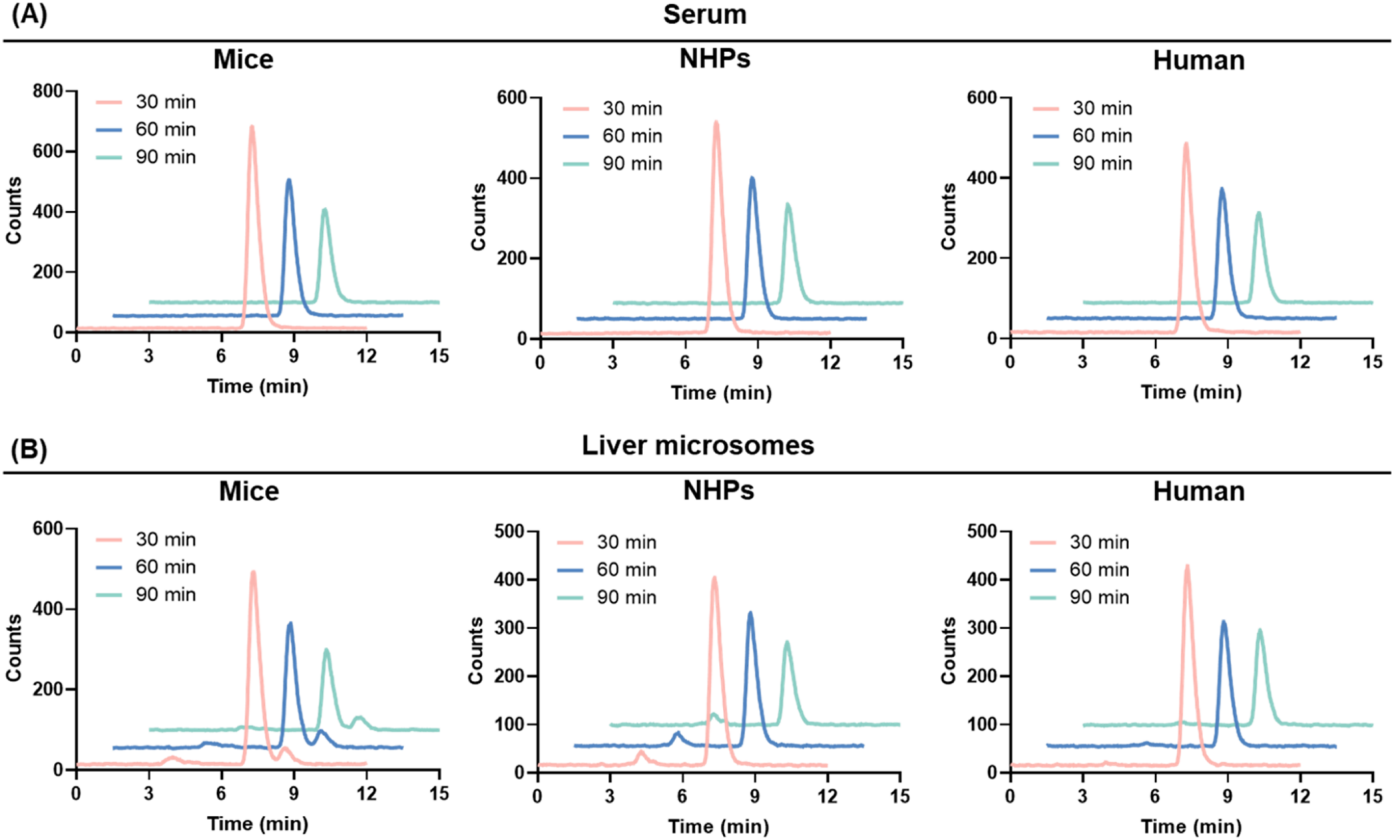
(A) Formulation stability of [^18^F]**3** in serum of mice, NHPs, and human at 30-, 60-, and 90 min; (B) Formulation stability of [^18^F]**3** in liver microsomes of mice, NHPs, and human at 30-, 60-, and 90 min.

Next, *ex vivo* stability in the brain and plasma of CD1 mice was analyzed by radio-HPLC (Figure 7). At 30 min postinjection, the parent fraction of [^18^F]**3** in the brain was determined to be 96%, indicating excellent *in vivo* metabolic stability. In the blood, 31% of the radioactivity remained intact. These findings suggest that [^18^F]**3** exhibited suitable stability for brain PET imaging applications.

**Figure 7:**
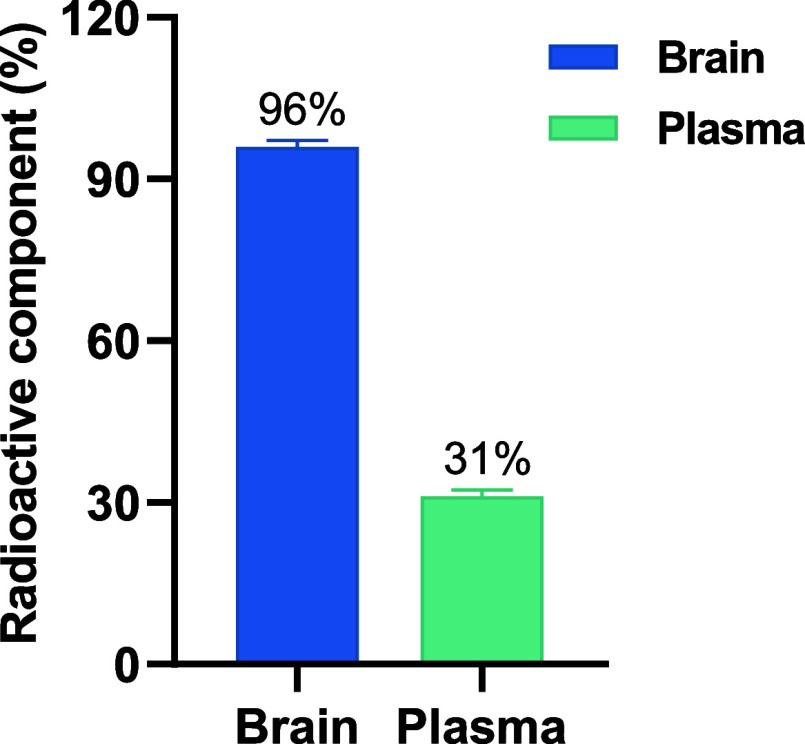
Radiometabolic stability in CD1 mice brain and plasma after injecting [^18^F]**3** for 30 min. All data are presented as mean ± SD, *n* = 2.

## Conclusions

Building on our previous work with H_3_R PET radioligands, [^18^F]­H3-2404 and [^11^C]­AZ13153556, we designed and synthesized several novel H_3_R antagonists. Initial SAR studies identified compounds **3** and **4** as promising H_3_R ligands, demonstrating high binding affinity (*K*_i_ = 2.87 and 3.15 nM, respectively) and selectivity against other CNS targets. Radiosynthesis of [^18^F]**3** ([^18^F]­H3-2406) and [^18^F]**4** ([^18^F]­H3-2407) were achieved with high radiopurity and molar activity. Both radioligands revealed heterogeneous regional distributions in the rat brain, consistent with known H_3_R expression patterns, as evidenced by *in vitro* autoradiography. Biodistribution and stability studies further confirmed that [^18^F]**3** possesses favorable performance characteristics, supporting its potential as a PET radioligand for noninvasive quantification of H_3_R. In PET imaging studies, [^18^F]**3** exhibited moderate brain uptake and gradual washout kinetics, whereas [^18^F]**4** lacked substantial *in vivo* specificity. Although [^18^F]**3** emerged as the most promising H_3_R PET ligand among the candidates, its specific binding to H_3_R was limited due to off-target binding to sigma-1 receptor. Our novel H_3_R PET ligand introduces a 4-cyclopentylpiperazin scaffold, which, to the best of our knowledge, has not been described before for H_3_R-targeted imaging. This unique scaffold offers several potential advantages, including enhanced metabolic stability and favorable physicochemical properties with good brain uptake (>10 %ID/g tissue, SUV of 2.4). While it is too early to predict whether this scaffold will overcome the current limitations, its structural diversity holds promise for addressing contemporary challenges in H_3_R PET tracer development. Future work will focus on further structural optimization of compound **3** to improve its selectivity over other CNS targets. Further, we will envision to optimize H_3_R PET ligands in disease-relevant mouse models, including those of neurodegenerative disorders such as Alzheimer’s disease and Parkinson’s disease, which are associated with H_3_R dysregulation.^49–51^

## Experimental Section

### General Information

The experimental procedures for compound synthesis and characterization were conducted as previous reports with minor modifications.^33,52^ All reagents and solvents were purchased from commercial sources (ChemShuttle and Sigma-Aldrich) without further purification. Thin-layer chromatography (TLC) analysis was performed by chemical HSGF254 silica gel plates (0.2 mm, Millipore Sigma). ^1^H NMR spectra were recorded on a 400 MHz Bruker spectrometer. ^13^C NMR spectra were recorded on a 100 MHz Bruker spectrometer. ^19^F NMR spectra were recorded on a 375 MHz Bruker spectrometer. Multiplicity of ^1^H NMR signals was reported as singlet (s), doublet (d), triplet (t), quartert (q), and multiplet (m), br (broad signal), dd (doublet of doublets), and so forth. Coupling constants (*J*) are reported in hertz (Hz). The melting point was determined using a Barnstead International-1101D melting point apparatus with a capillary tube method and is uncorrected. Mass spectra (MS) were recorded on a Shimadzu LC/MS-2020 spectrometer and high-resolution mass spectra (HRMS) data were recorded on a Thermo Fisher Scientific UPLC-ESI-Q-Orbitrap mass spectrometer in the ESI mode. The purification of all final compounds was performed by pre-HPLC (SHIMADZU LH-40) on Welch Xtimate C18 column (40 × 200 mm, 7 μm) with gradient mobile phases [water (NH_3_·H_2_O/NH_4_HCO_3_)-MeCN (acetonitrile), gradient: 0% to 66% MeCN over 25 min]. The purification of radioligands was performed by reverse-phase HPLC on Phenomenex Gemini C6-phenyl 110A column (10.0 × 250 mm, 5 μm). All final reference compounds are >95% purity, as confirmed by Shimadzu LC-20AD HPLC [mobile phase: ramp from 3% MeCN (0.02% TFA) in water (0.04% TFA) to 60% MeCN in water in 8.0 min; again from 60% MeCN (0.02% TFA) in water (0.04% TFA) to 80% MeCN in water in 2.0 min; return back to 3% MeCN (0.02%TFA) and hold for 3.0 min. Flow rate: 0.5 mL/min, column temperature: 40 °C, and detector wavelength at 220, 254, and 215 nm. Column: Halo C18 3.0 × 100 mm, 2.7 μm]. The purity of radioligands was confirmed by Agilent 1100 series HPLC system on Waters XSELECT HSS T3 column (4.6 × 150 mm, 5 μM). All animal studies were carried out in accordance with ethical guidelines of Institutional Animal Care and Use Committee (IACUC) of Emory University (IAMEND202400000623, protocol: PROTO202200003, PROTO202200076). CD-1 mice (Charles River lab, female, 22–24 g, 5–6 weeks, stain code: 022) and SD rats (Charles River lab, male, 210–230 g, 7–8 weeks, stain code: 001) were fed ad libitum with food and water under a condition of 12 h light/12 h dark cycle.

#### General Procedure for Synthesis of H_3_R Antagonists **1**–**7**

##### (4-Cyclopropylpiperazin-1-yl)­((1*S*,2*S*)-2-(4-fluorophenyl)­cyclopropyl)­methanone (**1**)

A mixture of commercial intermediate **10** (70.0 mg, 389 μmol), commercial intermediate **11a** (58.8 mg, 466 μmol), DIEA (251 mg, 1.94 mmol, 338 μL), HATU (177 mg, 466 μmol) in DMF (1.50 mL) was degassed and purged with N_2_ for 3 times, and then the mixture was stirred at 25 °C for 12 h under N_2_ atmosphere. The reaction mixture was diluted with EtOAc 20.0 mL and washed with H_2_O 60.0 mL (20.0 mL × 3). The combined organic layers were washed with brine 100 mL (50.0 mL × 2), dried over Na_2_SO_4_, filtered and concentrated under reduced pressure to give a residue. The residue was purified by prep-HPLC to give compound **1** (70.0 mg, 235 μmol, 75.4% yield, 97.0% purity) as a yellow solid. LC/MS: *m*/*z* = 289.2 (M + H)^+^. Melting point: 65–67 °C. ^1^H NMR (400 MHz, CDCl_3_) δ 7.02–7.14 (m, 2H), 6.89–7.02 (m, 2H), 3.55–3.66 (m, 4H), 2.61 (d, *J* = 4.4 Hz, 4H), 2.38–2.53 (m, 1H), 1.87–1.95 (m, 1H), 1.63–1.67 (m, 2H), 1.20–1.26 (m, 1H), 0.39–0.51 (m, 4H). ^13^C NMR (100 MHz, CDCl_3_) δ 170.21, 161.50 (*J* = 121 Hz) 136.57 (*J* = 1.6 Hz), 127.62 (2C, *J* = 4.0 Hz), 115.31 (2C, *J* = 10.6 Hz), 53.58, 52.93, 45.49, 42.14, 38.40, 24.65, 22.97, 15.98, 5.86 (2C). ^19^F NMR (375 MHz, CDCl3) δ 116.72. ESI-HRMS *m*/*z*: calcd for C_17_H_21_FN_2_O [M + H]^+^ 289.1711, found 289.1708.

Compounds **2**–**7** were synthesized following the same procedure as the one outlined for compound **1**. Key intermediates **11b**–**11e** are commercially available, the preparation of intermediates **11f** and **11g** is depicted in Supporting Information.

##### (4-Cyclobutylpiperazin-1-yl)­((1*S*,2*S*)-2-(4-fluorophenyl)­cyclopropyl)­methanone (**2**)

75.0% yield, 99.0% purity of white solid. LC/MS: *m*/*z* = 303.2­(M + H)^+^. Melting point: 70–72 °C. ^1^H NMR (400 MHz, MeOD) δ 7.12–7.22 (m, 2H), 6.93–7.07 (m, 2H), 3.59–3.77 (m, 4H), 2.78 (t, *J* = 7.6 Hz, 1H), 2.27–2.43 (m, 5H), 2.14–2.21 (m, 1H), 2.01–2.12 (m, 2H), 1.84–1.96 (m, 2H), 1.65–1.81 (m, 2H), 1.45–1.56 (m, 1H), 1.26–1.31 (m, 1H). ^13^C NMR (100 MHz, MeOD) δ 172.83, 163.15 (*J* = 121 Hz) 137.89, 129.10 (2C, *J* = 4.0 Hz), 116.30 (2C, *J* = 10.8 Hz), 61.10, 50.08, 50.03, 45.27, 41.97, 27.35 (2C), 26.02, 23.64, 16.94, 14.98. ^19^F NMR (375 MHz, MeOD) δ 119.03. ESI-HRMS *m*/*z*: calcd for C_18_H_23_FN_2_O [M + H]^+^ 303.1867, found 303.1864.

##### (4-Cyclopentylpiperazin-1-yl)­((1*S*,2*S*)-2-(4-fluorophenyl)­cyclopropyl)­methanone (**3**)

68.5% yield, 99.5% purity of white solid. LC/MS: *m*/*z* = 317.2 (M + H)^+^. Melting point: 86–87 °C. ^1^H NMR (400 MHz, CDCl_3_) δ 7.02–7.12 (m, 2H), 6.96 (s, 2H), 3.65 (br dd, *J* = 10, 4.8 Hz, 4H), 2.49 (br d, *J* = 6.0 Hz, 5H), 1.82–1.93 (m, 3H), 1.67–1.74 (m, 2H), 1.62–1.65 (m, 2H), 1.56 (br dd, *J* = 7.6, 4.8 Hz, 2H), 1.35–1.44 (m, 2H), 1.22 (ddd, *J* = 8.0, 6.4, 4.8 Hz, 1H). ^13^C NMR (100 MHz, CDCl_3_) δ 170.10, 161.49­(*J* = 243 Hz) 136.58 (*J* = 3.0 Hz), 127.59 (2C, *J* = 7.0 Hz), 115.30 (2C, *J* = 22.0 Hz), 67.33, 52.55, 51.87, 45.50, 42.14, 30.34 (2C), 24.63, 24.07 (2C), 22.90, 15.99. ESI-HRMS *m*/*z*: calcd for C_19_H_25_FN_2_O [M + H]^+^ 317.2024, found 317.2019.

##### (4-Cyclohexylpiperazin-1-yl)­((1*S*,2*S*)-2-(4-fluorophenyl)­cyclopropyl)­methanone (**4**)

73.1% yield, 99.8% purity of white solid. LC/MS: *m*/*z* = 331.4 (M + H)^+^. Melting point: 81–83 °C. ^1^H NMR (400 MHz, CDCl_3_) δ 7.08–7.10 (m, 2H), 6.94–7.01 (m, 2H), 3.60–3.67 (m, 4H), 2.54–2.59 (m, 4H), 2.45–2.51 (m, 1H), 2.25–2.33 (m, 1H), 1.88–1.94 (m, 1H), 1.78–1.87 (m, 4H), 1.64 (dt, *J* = 8.8, 4.6 Hz, 2H), 1.18–1.27 (m, 5H), 1.08–1.17 (m, 1H). ^13^C NMR (100 MHz, CDCl_3_) δ 170.09, 162.49 (*J* = 243 Hz), 136.65 (*J* = 3.0 Hz),127.61 (*J* = 7.0 Hz), 115.29 (*J* = 21.0 Hz), 63.58, 49.40, 48.65, 46.08, 42.71, 28.88 (2C), 26.25, 25.82 (2C), 24.60, 22.92, 15.96. ESI-HRMS *m*/*z*: calcd for C_20_H_27_FN_2_O [M + H]^+^ 331.2180, found 331.2175.

##### (4-Cycloheptylpiperazin-1-yl)­((1*S*,2*S*)-2-(4-fluorophenyl)­cyclopropyl)­methanone (**5**)

62.0% yield, 98.3% purity of white solid. LC/MS: *m*/*z* = 345.1 (M + H)^+^. Melting point: 73–75 °C. ^1^H NMR (400 MHz, CDCl_3_) δ 7.04–7.11 (m, 2H), 6.93–7.02 (m, 2H), 3.71 (br s, 4H), 3.00–3.46 (m, 1H), 2.61–2.75 (m, 5H), 2.45–2.52 (m, 1H), 1.60–1.90 (m, 6H), 1.38–1.56 (m, 8H). ^13^C NMR (100 MHz, CDCl_3_) δ 170.26, 161.52 (*J* = 121 Hz) 136.40 (*J* = 1.6 Hz), 127.61 (2C, *J* = 4.0 Hz), 115.33 (2C, *J* = 10.7 Hz), 65.50, 48.71, 47.81, 45.16, 41.75, 29.46 (2C), 27.85 (2C), 25.34 (2C), 24.77, 22.78, 16.18. ^19^F NMR (375 MHz, CDCl_3_) δ 116.66. ESI-HRMS *m*/*z*: calcd for C_21_H_29_FN_2_O [M + H]^+^ 345.2337, found 345.2329.

##### (6-Cyclobutyl-2,6-diazaspiro­[3.3]­heptan-2-yl)­((1*S*,2*S*)-2-(4-fluorophenyl)­cyclopropyl)­methanone (**6**)

49.7% yield, 98.2% purity of white solid. LC/MS: *m*/*z* = 315.2 (M + H)^+^. Melting point: 73–75 °C. ^1^H NMR (400 MHz, CDCl_3_) δ 7.02–7.09 (m, 2H), 6.89–7.01 (m, 2H), 4.25–4.34 (m, 2H), 4.07 (s, 2H), 3.26–3.33 (m, 4H), 3.05 (br t, *J* = 7.6 Hz, 1H), 2.40–2.52 (m, 1H), 1.87–1.99 (m, 3H), 1.79 (br d, *J* = 9.6 Hz, 1H), 1.62–1.74 (m, 2H), 1.53–1.60 (m, 2H), 1.15–1.24 (m, 1H). ^13^C NMR (100 MHz, CDCl_3_) δ 171.90, 161.50 (*J* = 121 Hz), 136.38 (*J* = 1.6 Hz), 127.66 (2C, *J* = 4.0 Hz), 115.26 (2C, *J* = 10.7 Hz), 60.33, 60.31, 60.27, 59.93, 58.01, 32.83, 25.17 (2C), 24.52, 21.84, 16.02, 14.71. ^19^F NMR (375 MHz, CDCl_3_) δ 116.66. ESI-HRMS *m*/*z*: calcd for C_19_H_23_FN_2_O [M + H]^+^ 315.1867, found 315.1863.

##### ((1*S*,4*S*)-5-Cyclobutyl-2,5-diazabicyclo­[2.2.1]­heptan-2-yl)­((1*S*,2*S*)-2-(4-fluorophenyl)­cyclopropyl)­methanone (**7**)

40.6% yield, 99.8% purity of white solid. LC/MS: *m*/*z* = 315.2 (M + H)^+^. Melting point: 61–64 °C. ^1^H NMR (400 MHz, CDCl_3_) δ 7.04–7.10 (m, 2H), 6.94–7.01 (m, 2H), 4.33–4.84 (m, 1H), 3.39–3.68 (m, 3H), 2.93–3.31 (m, 2H), 2.72–2.81 (m, 1H), 2.42–2.67 (m, 2H), 1.98–2.07 (m, 2H), 1.74–1.94 (m, 6H), 1.12–1.40 (m, 2H). ^19^F NMR (375 MHz, CDCl_3_) δ 116.82. ESI-HRMS *m*/*z*: calcd for C_19_H_23_FN_2_O [M + H]^+^ 315.1867, found 315.1863.

#### General Procedure for Synthesis of H_3_R Antagonist **8**

##### *tert*-Butyl (3*aR*,6*aS*)-5-((1*S*,2*S*)-2-(4-Fluorophenyl)­cyclopropane-1-carbonyl)­hexahydropyrrolo­[3,4-*c*]­pyrrole-2­(1H)-carboxylate (**13**)

To a solution of commercial intermediate **10** (200 mg, 1.11 mmol) in DMF (5.00 mL) was added HATU (506 mg, 1.33 mmol), DIEA (717 mg, 5.55 mmol, 967 μL) and commercial intermediate **12** (353 mg, 1.67 mmol). The mixture was stirred at 25 °C for 12 h under N_2_ atmosphere. The reaction solution diluted with H_2_O (10.0 mL) and extracted with EtOAc 30.0 mL (10.0 mL × 3) and brine 20.0 mL (10.0 mL × 2). The organic layer was dried (Na_2_SO_4_) and concentrated in vacuo to afford crude compound **13** (480 mg, crude) as a yellow oil. LC/MS: *m*/*z* = 397.1 (M+Na)^+^.

##### ((1*S*,2*S*)-2-(4-Fluorophenyl)­cyclopropyl)­((3*aR*,6*aS*)-hexahydropyrrolo­[3,4-*c*]­pyrrol-2­(1H)-yl)­methanone (**14**)

To a solution of compound **13** (480 mg, 1.28 mmol) in DCM (6.00 mL) was added TFA (3.07 g, 26.9 mmol, 2.00 mL). The mixture was stirred at 25 °C for 12 h under N_2_ atmosphere. The reaction mixture was filtered and concentrated under reduced pressure to give crude compound **14** (640 mg, crude TFA salt) as a yellow solid. LC/MS: *m*/*z* = 275.2 (M + H)^+^.

##### ((3*aR*,6*aS*)-5-Cyclobutylhexahydropyrrolo­[3,4-*c*]­pyrrol-2­(1H)-yl)­((1*S*,2*S*)-2-(4-fluorophenyl)­cyclopropyl)­methanone (**8**)

To a solution of compound **14** (200 mg, 540 μmol) in MeOH (5.00 mL) adjust pH > 7 of reaction solution with TEA (73.8 mg, 729 μmol, 101 μL). Compound **15** (76.6 mg, 1.09 mmol, 81.7 μL) and AcOH (21.9 mg, 364 μmol, 20.9 μL) were added and stirred 1 h at 25 °C. Degassed and purged with N_2_ 3 times and in batches under 0 °C NaBH_3_CN (137 mg, 2.19 mmol) was added. The mixture was stirred at 25 °C for 11 h under N_2_ atmosphere. The residue was diluted with H_2_O (10.0 mL) and extracted with EtOAc 60.0 mL (20.0 mL × 3). The combined organic layers were washed with brine 40.0 mL (20.0 mL × 2), dried over Na_2_SO_4_, filtered and concentrated under reduced pressure to give a residue. The residue was purified by prep-HPLC to give compound **8** (31.0 mg, 94.2 μmol, 12.9% yield, 99.8% purity) as a white solid. LC/MS: *m*/*z* = 329.1 (M + H)^+^. Melting point: 101–103 °C. ^1^H NMR (400 MHz, CDCl_3_) δ 7.06 (br d, *J* = 5.6 Hz, 2H), 6.96 (br d, *J* = 4.0 Hz, 2H), 3.72–3.83 (m, 1H), 3.54–3.69 (m, 3H), 3.30 (br s, 4H), 3.07 (br s, 1H), 2.51–2.61 (m, 2H), 2.26–2.38 (m, 2H), 2.13 (br d, *J* = 7.2 Hz, 2H), 1.83–1.93 (m, 1H), 1.71–1.82 (m, 2H), 1.59–1.66 (m, 1H), 1.22–1.31 (m, 2H). ^19^F NMR (375 MHz, CDCl_3_) δ 116.52.

#### General Procedure for Synthesis of H_3_R Antagonist **9**

##### ((1*S*,2*S*)-2-(4-Bromophenyl)­cyclopropyl)­(4-cyclobutylpiperazin-1-yl)­methanone (**17**)

To a solution of commercial intermediate **16** (200 mg, 830 μmol) in DMF (3.00 mL) was added HATU (379 mg, 996 μmol), DIEA (536 mg, 4.15 mmol, 723 μL) and commercial intermediate **11b** (140 mg, 996 μmol) was degassed and purged with N_2_ for 3 times, and then the mixture was stirred at 25 °C for 18 h under N_2_ atmosphere. The residue was diluted with water (20.0 mL) and extracted with EtOAc 60.0 mL (20.0 mL × 3), dried over Na_2_SO_4_, filtered and concentrated under reduced pressure to give a residue. The residue was purified by flash silica gel chromatography (eluent of 0 ∼10.0% dichloromethane: methanol, 20.0 mL/min) to afford compound **17** (343 mg, crude) as a white solid. LC/MS: *m*/*z* = 364.9 (M + H)^+^.

##### Methyl 4-((1*S*,2*S*)-2-(4-cyclobutylpiperazine-1-carbonyl)­cyclopropyl)­benzoate (**18**)

To a solution of compound **17** (300 mg, 826 μmol) in MeOH (10.0 mL) was added TEA (251 mg, 2.48 mmol, 345 μL) and Pd­(dppf)­Cl_2_ (120.85 mg, 165 μmol) under N_2_. The mixture was stirred at 80 °C for 60 h under CO (50 psi). The reaction mixture was filtered and concentrated under reduced pressure to give compound **18** (240 mg, 622 μmol, 75.4% yield, 88.8% purity) as a yellow solid. LC/MS: *m*/*z* = 343.3 (M + H)^+^.

##### 4-((1*S*,2*S*)-2-(4-Cyclobutylpiperazine-1-carbonyl)­cyclopropyl)­benzoic acid (**19**)

To a solution of compound **18** (200 mg, 584 μmol) in THF (2.00 mL) and MeOH (1.00 mL) was added a solution of LiOH·H_2_O (270 mg, 6.42 mmol) in H_2_O (0.5 mL). Then the mixture was stirred at 25 °C for 5 h under N_2_. The reaction mixture was added 1 M HCl to make pH = 2, then diluted with EtOAc 20.0 mL and washed with H_2_O 60.0 mL (20.0 mL × 3). The combined organic layers were washed with brine 100 mL (50.0 mL × 2), dried over Na_2_SO_4_, filtered and concentrated under reduced pressure to give a compound **19** (140 mg, 355 μmol, 60.7% yield, 83.2% purity) as a yellow solid. LC/MS: *m*/*z* = 329.1 (M + H)^+^.

##### (4-Cyclobutylpiperazin-1-yl)­((1*S*,2*S*)-2-(4-(3-fluoroazetidine-1-carbonyl)­phenyl)­cyclopropyl)­methanone (**9**)

A mixture of compound **19** (50.0 mg, 152 μmol), compound **20** (34.0 mg, 305 μmol, HCl), HATU (69.5 mg, 183 μmol), DIEA (98.4 mg, 761 μmol, 133 μL) in DMF (2.00 mL) was degassed and purged with N_2_ for 3 times, and then the mixture was stirred at 25 °C for 3 h under N_2_ atmosphere. The reaction mixture was diluted with EtOAc 20.0 mL and washed with H_2_O 60.0 mL (20.0 mL × 3). The combined organic layers were washed with brine 100 mL (50.0 mL × 2), dried over Na_2_SO_4_, filtered and concentrated under reduced pressure to give a residue. The residue was purified by prep-HPLC to give compound **9** (24.6 mg, 63.7 μmol, 41.8% yield, 99.8% purity) as a white solid. LC/MS: *m*/*z* = 329.1 (M + H)^+^. Melting point: 149–151 °C. ^1^H NMR (400 MHz, CDCl_3_) δ 7.55 (d, *J* = 8.0 Hz, 2H), 7.14 (d, *J* = 8.4 Hz, 2H), 5.19–5.54 (m, 1H), 4.44–4.54 (m, 2H), 4.21–4.42 (m, 2H), 3.50–3.82 (m, 4H), 2.60–2.89 (m, 1H), 2.45–2.57 (m, 1H), 2.32 (s, 4H), 1.97–2.11 (m, 3H), 1.79–1.96 (m, 2H), 1.64–1.76 (m, 3H), 1.26–1.33 (m, 1H). ^13^C NMR (100 MHz, CDCl_3_) δ 170.34, 169.84, 144.91, 130.60, 128.19 (2C), 126.04 (2C), 83.27, 81.23, 60.02 (2C), 49.69, 49.00, 45.33, 41.96, 26.96 (2C), 25.17, 23.57, 16.56, 14.27. ^19^F NMR (375 MHz, CDCl_3_) δ 180.32. ESI-HRMS *m*/*z*: calcd for C_22_H_28_FN_3_O_2_ [M + H]^+^ 386.2238, found 386.2233.

#### General Procedure for Synthesis of Precursor **22**

##### ((1*S*,2*S*)-2-(4-Bromophenyl)­cyclopropyl)­(4-cyclopentylpiperazin-1-yl)­methanone (**21**)

A mixture of compound **16** (200 mg, 829 μmol), DIEA (536 mg, 4.15 mmol), T3P (1.79 g, 2.49 mmol, 50% purity) in CH_2_Cl_2_ (2.00 mL) and compound **11c** (192 mg, 1.24 mmol) was added in, and then the mixture was stirred at 25 °C for 2 h. The reaction mixture was diluted with H_2_O (5.00 mL) and extracted with CH_2_Cl_2_ 30.0 mL (10.0 mL × 3). The combined organic layers were concentrated under reduced pressure to afford compound **21** (420 mg, crude) as a white solid. LC/MS: *m*/*z* = 378.8 (M + H)^+^.

##### (4-Cyclopentylpiperazin-1-yl)­((1*S*,2*S*)-2-(4-(4,4,5,5-tetramethyl-1,3,2-dioxaborolan-2-yl)­phenyl)­cyclopropyl)­methanone (**22**)

A mixture of compound **21** (400 mg, 1.06 mmol), BPD (538 mg, 2.12 mmol), Pd­(dppf)­Cl_2_ (155 mg, 212 μmol), AcOK (312 mg, 3.18 mmol) in dioxane (5.00 mL) was degassed and purged with N_2_ for 3 times, and then the mixture was stirred at 100 °C for 3 h under N_2_ atmosphere. The reaction mixture was filtered and concentrated under reduced pressure to give a residue. The residue was purified by flash silica gel chromatography (eluent of 0∼56% ethyl acetate/dichloromethane gradient@25 mL/min) to afford compound **22** (120 mg, 283 μmol, 26.7% yield, 99.9% purity) as a pink solid. LC/MS: *m*/*z* = 425.3 (M + H)^+^. Melting point: 155–156 °C. ^1^H NMR (400 MHz, CDCl_3_) δ 7.73 (d, *J* = 8.0 Hz, 2H), 7.11 (d, *J* = 8.0 Hz, 2H), 3.55–3.76 (m, 4H), 2.42–2.54 (m, 6H), 1.95–2.02 (m, 1H), 1.81–1.91 (m, 2H), 1.67–1.73 (m, 3H), 1.63 (br s, 1H), 1.55–1.58 (m, 1H), 1.37–1.45 (m, 2H), 1.34 (s, 12H), 1.31 (dt, *J* = 6.4, 1.6 Hz, 1H). ^13^C NMR (100 MHz, CDCl_3_) δ 170.08, 144.44, 135.01 (2C), 125.33 (2C), 83.75 (2C), 67.32, 52.54, 51.87, 45.48, 42.11, 30.32 (2C), 25.58, 24.85 (4C), 24.07 (2C), 23.53, 16.27. ESI-HRMS *m*/*z*: calcd for C_25_H_37_BN_2_O_3_ [M + H]^+^ 425.2970, found 425.2970.

#### General Procedure for Synthesis of Precursor **24**

##### ((1*S*,2*S*)-2-(4-Bromophenyl)­cyclopropyl)­(4-cyclohexylpiperazin-1-yl)­methanone (**23**)

A mixture of compound **16** (200 mg, 829 μmol), DIEA (536 mg, 4.15 mmol), T3P (1.79 g, 2.49 mmol, 50% purity) and compound **11d** (167 mg, 995 μmol) in CH_2_Cl_2_ (2.00 mL), was degassed and purged with N_2_ for 3 times, and then the mixture was stirred at 25 °C for 2h under N_2_ atmosphere. The reaction mixture was diluted with H_2_O (5.00 mL) and extracted with CH_2_Cl_2_ 30.0 mL (10.0 mL × 3). The combined organic layers were concentrated under reduced pressure to afford compound **23** (380 mg, crude) as a white solid. LC/MS: *m*/*z* = 392.8 (M + H)^+^.

##### (4-Cyclohexylpiperazin-1-yl)­((1*S*,2*S*)-2-(4-(4,4,5,5-tetramethyl-1,3,2-dioxaborolan-2-yl)­phenyl)­cyclopropyl)­methanone (**24**)

A mixture of compound **23** (360 mg, 920 μmol), BPD (467 mg, 1.84 mmol), Pd­(dppf)­Cl_2_ (135 mg, 184 μmol), AcOK (270 mg, 2.76 mmol) in dioxane (6.00 mL) was degassed and purged with N_2_ for 3 times, and then the mixture was stirred at 100 °C for 3h under N_2_ atmosphere. The reaction mixture was filtered and concentrated under reduced pressure to give a residue. The residue was purified by flash silica gel chromatography (eluent of 0∼50% ethyl acetate/dichloromethane gradient@25 mL/min) to afford compound **24** (110 mg, 236 μmol, 25.6% yield, 93.88% purity) as a brown solid. LC/MS: *m*/*z* = 439.3 (M + H)^+^. Melting point: 92–95 °C. ^1^H NMR (400 MHz, CDCl_3_) δ 7.73 (d, *J* = 8.0 Hz, 2H), 7.11 (d, *J* = 8.0 Hz, 2H), 3.56–3.70 (m, 4H), 2.51–2.60 (m, 4H), 2.44–2.50 (m, 1H), 2.24–2.34 (m, 1H), 1.96–2.01 (m, 1H), 1.74–1.87 (m, 6H), 1.62–1.71 (m, 2H), 1.34 (s, 12H), 1.29–1.32 (m, 1H), 1.25–1.28 (m, 1H), 1.23 (br s, 1H), 1.21 (s, 1H). ^13^C NMR (100 MHz, CDCl_3_) δ 170.07, 144.47, 135.00 (2C), 125.34 (2C), 83.75 (2C), 63.64, 49.34, 48.60, 45.91, 42.54, 28.76 (2C), 26.21, 25.80 (2C), 25.57, 24.85 (4C), 23.54, 16.26. ESI-HRMS *m*/*z*: calcd for C_26_H_39_BN_2_O_3_ [M + H]^+^ 439.3126, found 439.3124.

### Radiosynthesis of H_3_R Radioligands [^18^F]**3** and [^18^F]**4**

The [^18^F]­fluoride (1.1 GBq) was trapped by a Sep-Pak QMA Plus Light cartridge (Waters) and subsequently eluted into a reaction vessel with a solution of TEAB (1 mg) in MeOH (0.1 mL) and acetonitrile (1 mL). The reaction mixture was dried three times using acetonitrile (1 mL each) at 110 °C under a N_2_ atmosphere. Precursors **22** or **24** (1.5 mg) and Cu­(OTf)_2_Py_4_ (8 mg) in anhydrous DMA/*n*-BuOH (0.2/0.1 mL) were then added and heated at 120 °C for 15 min. After dilution with H_2_O (10 mL), the reaction mixture was trapped on a Sep-Pak light C18 cartridge (Waters). The crude product was eluted with acetonitrile (1 mL) into H_2_O (3 mL), and the mixture was injected into pre-HPLC (Lablogic HPLC, Phenomenex Luna C-18 column [250 × 10 mm, 5 μm], mobile phase 35% and 37% CH_3_CN/0.1% TEA in water, flow:5 mL/min). The radioactive [^18^F]**3** or [^18^F]**4** fractions were collected in a sterile flask with retention time of 22 and 50 min, respectively. These fractions were diluted with H_2_O (40 mL) and trapped on a Sep-Pak light C18 cartridge (Waters). The products were washed from the cartridge with ethanol (0.3 mL) and formulated with PBS (3 mL). Radiochemical purity was assessed via analytical HPLC (Agilent 1100 series HPLC system on Phenomenex Luna C-18 column (4.6 × 150 mm), mobile phase 25% or 40% CH_3_CN/0.1% TFA in water, flow:1 mL/min). The nondecay corrected radiochemical yields of [^18^F]**3** and [^18^F]**4** were determined to be 32.4 and 22.6%, respectively. The purities of [^18^F]**3** and [^18^F]**4** were determined to be >99% with molar activities of 103 GBq/μmol and 30 GBq/μmol, respectively. The identification of two radioligands was confirmed by coinjection with unlabeled compounds.

### H_3_R Antagonists Binding Assay

H_3_R antagonists binding assay was performed using a competitive [^3^H]­AZ13582963 ligands by Gifford Bioscience following published protocols (https://www.giffordbioscience.com/wp-content/uploads/2024/06/Radioligand-Binding-Assay-Protocol-document-compressed.pdf). [^3^H]­AZ13582963 ligands were prepared by AstraZeneca. LC/MS (M + H)^+^: 341 (27%), 343 (73%). ^3^H NMR (533 MHz, CD_3_OD): 7.31 (br, s). The radiochemical purity was >99.1% (Column: Waters XBridge C18 3.5 μm OBD 4.6 x100 mm, A: water/10 mmol NH_4_OH buffered to pH 10, B: MeCN, Gradient: B 5% for 3 min, then to 95% over 22 min and hold 95% for 5 min, flow rate: 1 mL/min). The specific activity was determined to be 769 GBq/mmol.

### Measurement of Log *D*

The lipophilicity was measured according to previously reported methods.^33,53^ In brief, PBS and n-octanol were premixed prior to the experiment. A solution containing 0.2 MBq of [^18^F]**3** or [^18^F]**4**, PBS (3 mL) and n-octanol (3 mL) were prepared in a vial and vortexed for 5 min. The PBS and n-octanol layers were then separated and transferred into a centrifuge tube, followed centrifugation at 12,000 rpm for 5 min. Then PBS (0.5 mL) and *n*-octanol (0.05 mL) were weight and measured the radioactivity on a γ counter (PerkinElmer Wizard). The Log *D* values of **[** ^18^F]**3** and [^18^F]**4** were calculated using the following formula:

$$\mathrm{Log}D=\mathrm{Log}\left[\left({\mathrm{radioactivity}}_{n‐\mathrm{octanol}}/{\mathrm{weight}}_{n‐\mathrm{octanol}}\right)/\left({\mathrm{radioactivity}}_{\mathrm{PBS}}/{\mathrm{weight}}_{\mathrm{PBS}}\right)\right]$$

### *In Vitro* ARG Studies of [^18^F]**3** and [^18^F]**4**

The ARG was measured according to previously reported methods.^54,55^ The brains of SD rat were sectioned into 15 μm slices and stored in −80 °C. For each experiment, the brain slices allowed them to warm to room temperature and then incubated in Tris-HCl buffer (50 mM, containing 0.1% bovine serum albumin) for 10 min. The slices were incubated with [^18^F]**3** or [^18^F]**4** (0.037 MBq/mL) in the presence of either DMSO (baseline study), GSK189254 (10 μM, blocking study), and unlabeled **3** or **4** (10 μM, blocking study) at room temperature for 30 min. Following incubation, the brain slices were washed three times with cold Tris-HCl buffer (50 mM) for 5 min each, and then dipped in water 2 times for 5 s. The brain slices were dried under cold air and exposed to a Phosphor Screen (BAS IP MS2025, Cytiva) for 2 h. The radioactivity and imaging were captured using an Amersham Typhoon 5 (Cytiva) and the data were analyzed using ImageJ software.

### *In Vivo* PET Imaging Studies of [^18^F]**3** and [^18^F]**4**

PET imaging was conducted following previously reported methods.^33^ CD1 mice (5–6 weeks) were anesthetized with 1.5% (v/v) isoflurane during the scan. A dose of 3.0 MBq of [^18^F]**3** or [^18^F]**4** was intravenously injected into the tail vein, and a dynamic scan was acquiring using a PET scanner (MOLECUBES, beta-cube) for 60 min. In the blocking studies, GSK189254 (3 mg/kg) in 0.1 mL of saline containing 5% ethanol and 5% Tween-80 was preinjected for 10 min prior to the injection of PET radioligands. The results were reconstructed and analyzed by PMOD 4.3 software.

### *Ex Vivo* Whole-Body Biodistribution of [^18^F]**3**

A dose of 1.0 MBq of [^18^F]**3** in 0.1 mL saline was intravenously injected into the tail vein of CD1 mice (5–6 weeks). At various time-points (5-, 15-, 30- and 60 min) postinjection, the mice were sacrificed, and their organs were collected and weighed. The radioactivity of each organ was measured using a γ counter (PerkinElmer Wizard).

### *In Vitro* Stability of [^18^F]**3** in Serum and Microsomes

Serum from mice, NHPs, and humans were warmed to room temperature, 15 MBq of [^18^F]**3** was added to 0.4 mL of each species’ serum. The mixtures were incubated at 37 °C and 0.1 mL of mixture was transferred into 0.2 mL cold acetonitrile at 30, 60, and 90 min. The samples were vortexed for 15 s and then centrifuged at 10,000*g* for 5 min. The supernatant was analyzed using analytical HPLC (Agilent 1100 series HPLC system on Phenomenex Luna C-18 column (4.6 × 150 mm), mobile phase 30% CH_3_CN/0.1% TFA in water, flow:1 mL/min). For microsomes studies, a mixture of microsomes (0.5 mg/mL), [^18^F]**3** (15 MBq), 0.35 mL of PBS, 0.05 mL of NADPH regenerating system A, and 0.01 mL of NADPH regenerating system B were incubated at 37 °C. The subsequent steps followed the same procedure as described above.

### *Ex Vivo* Metabolite Analysis of [^18^F]**3**

A dose of 15 MBq of [^18^F]**3** in 0.1 mL saline was intravenously injected into the tail vein of CD1 mice (5–6 weeks). The mice were sacrificed at 30 min postinjection, and the mice brain and blood were quickly collected and homogenized with cold acetonitrile. The samples were centrifuged, and the supernatants were separated using the pre-HPLC (Lablogic HPLC, Phenomenex Luna C-18 column [250 × 10 mm, 5 μm], mobile phase 50% CH_3_CN/0.1% TEA in water, flow:5 mL/min) by collecting the mobile phase eluent every 30 s for 15 min. The radioactivity for each fraction was measured using a γ counter (PerkinElmer Wizard). Data analysis was performed by GraphPad Prism 8.0, and the percentage of [^18^F]**3** relative to total radioactivity (corrected for decay) was calculated as (peak area for [^18^F]**3** /total peak area) × 100%.

## Supplementary Material





## Notes

The synthesis of intermediates **11f** and **11g**, characterization of designed H_3_R antagonists and supporting figures (PDF)
Molecular formula strings and some data (CSV)

## Abbreviations Used

AUC: area under curve
ARG: autoradiography
BBB: brain-blood barrier
BCRP: breast cancer resistance protein
CB1: cannabinoid receptors 1
CNS: central nervous system
GPCRs: G protein-coupled receptors
HATU: 1-[bis­(dimethylamino)­methylene]-1*H*-1,2,3-triazolo-[4,5-*b*]­pyridinium 3-oxide hexafluorophosphate
H_3_R: histamine subtype 3 receptor
HRMS: high-resolution mass spectra
5-HT2A: 5-hydroxytryptamine receptor 2A
IACUC: institutional animal care and use committee
(IDIF): image-derived input function
MPO: multiparameter optimization score
MS: mass spectra
NHPs: nonhuman primates
PET: positron emission tomography
P-gp: P-glycoprotein
RCY: radiochemical yield
SAR: structure–activity relationships
SUV: standardized uptake value
TACs: time-activity curves
TLC: thin-layer chromatography
*V*_T_: volume of distribution
