# Supplementary material for: Development and Preclinical Characterization of [18F]H3-2406 and [18F]H3-2407 for Positron Emission Tomography Imaging of the Histamine Subtype‑3 Receptor
Source: J Med Chem. 2025 Jul 16;68(15):15372–85. doi: 10.1021/acs.jmedchem.4c02924 (PMC12362585; doi:10.1021/acs.jmedchem.4c02924)

Supporting Information

Development and Preclinical Characterization of [^{18}F]H3-2406 and [^{18}F]H3-2407 for Positron Emission Tomography Imaging of the Histamine Subtype-3 Receptor

Zhendong Song[a,§], Yinlong Li[a,§], Kenneth Dahl[b,c], Zhenkun Sun[d], Jiahui Chen[a], Xin Zhou[a], Yabiao Gao[a], Jian Rong[a], Chunyu Zhao[a], Katherine Yuan[a], Ahmad F. Chaudhary[a], Jimmy S. Patel[a,e], Thomas L. Collier[a], Chongzhao Ran[f], Kim S. Muehlfenzl[g], Achi Haider[a], Charles S. Elmore[g], Magnus Schou[b,c,]*, Steven H. Liang[a,]*

[a]*Department of Radiology and Imaging Sciences, Emory University, 1364 Clifton Road, Atlanta, GA, 30322, USA.*

[b]*PET Science Centre, Precision Medicine and Biosamples, Oncology R&D, AstraZeneca, Karolinska Institutet, Stockholm, 17176, Sweden.*

[c]*Department of Clinical Neuroscience, Centre for Psychiatry Research, Karolinska Institutet and Stockholm County Council, Stockholm, 17176, Sweden.*

[d]*Department of Pharmacology and Chemical Biology, Emory University School of Medicine, Atlanta, GA, 30322, USA.*

[e]*Department of Radiation Oncology, Winship Cancer Institute of Emory University, Atlanta, GA, 30322, USA.*

[f]*Athinoula A. Martinos Center for Biomedical Imaging, Department of Radiology, Massachusetts General Hospital and Harvard Medical School, Boston, Massachusetts, 02114, USA.*

[g]*Early Chemical Development, Pharmaceutical Sciences, R&D, AstraZeneca Pharmaceuticals, Gothenburg, 43183, Sweden.*

[§]These authors contributed equally to this work.

[*]Corresponding Authors

[*]Magnus Schou, E-mail: magnus.schou@astrazeneca.com; [*]Steven H. Liang, E-mail: steven.liang@emory.edu

Contents

General Procedure for Synthesis of 11f and 11g 3

Figure S1. Off-target pharmacological evaluation of compound 2, compound 3, and compound 4 4

Figure S2. The binding affinities between reference compounds and CNS targets. 5

Figure S3. TACs of [^{18}F]3 in the whole-brain under baseline blocking conditions. 5

Figure S4. Mice PET imaging studies (0−60 minutes) of [^{18}F]3 6

Figure S5. Molar activity 6

Table S1. Estimation of V_T values by Logan analysis in mice with [^{18}F]3................................7

^{1}H NMR, ^{13}C NMR, and ^{19}F NMR spectra 8

HPLC analysis of Compounds 3 and 4 22

Semi-prep HPLC of Radioligands [^{18}F]3 and [^{18}F]4 24

Analytical-HPLC of Radioligands [^{18}F]3 and [^{18}F]4 25

General Procedure for Synthesis of 11f and 11g

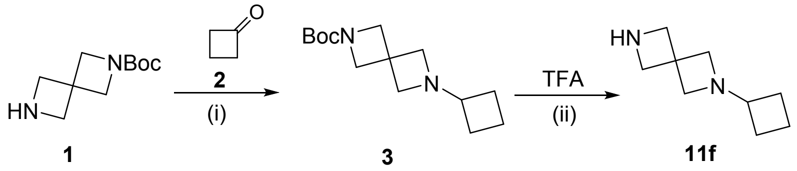

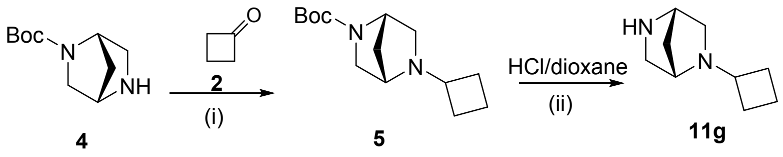

To a solution of compound **1** (100 mg, 1.43 mmol, 1.00 eq), compound **2** (411 mg, 1.43 mmol, 1.00 eq) in MeOH (2.00 mL) was added TEA (173 mg, 1.71 mmol, 1.20 eq) and $NaBH_3CN$ (269 mg, 4.28 mmol, 3.00 eq). The mixture was stirred at 25 °C for 12 h. The reaction mixture was diluted with EtOAc 50.0 mL and washed with H_2O 30.0 mL. Then the organic phase was washed with brine 30.0 mL (15.0 mL × 2), dried over Na_2SO_4, filtered and concentrated under reduced pressure to give a residue. The residue was purified by flash silica gel chromatography (ISCO®; 20 g SepaFlash® Silica Flash Column, Eluent of 0~10% MeOH/DCM @ 30 mL/min) to give compound **3** (250 mg, 991 μmol, 69.4% yield) as a yellow oil. LC/MS: m/z = 253.2 $(M+H)^+$.

To a solution of compound **3** (250 mg, 991 μmol, 1.00 eq) in DCM (3.00 mL) was added TFA (1.00 mL). The mixture was stirred at 25 °C for 12 h. The reaction mixture was concentrated under reduced pressure to give compound **11f** (300 mg, crude, TFA salt) as a yellow oil. LC/MS: m/z = 153.2 $(M+H)^+$.

To a solution of compound **4** (150 mg, 2.14 mmol, 1.50 eq), compound **2** (283 mg, 1.43 mmol, 1.00 eq) in MeOH (3.00 mL) was added $NaBH_3CN$ (269 mg, 4.28 mmol, 3.00 eq). The mixture was stirred at 25 °C for 12 h. The reaction mixture was diluted with EtOAc 50.0 mL and washed with H_2O 30.0 mL. Then the organic phase was washed with brine 30.0 mL (15.0 mL *2), dried over Na_2SO_4, filtered and concentrated under reduced pressure to give a residue. The residue was purified by flash silica gel

chromatography (ISCO®; 20 g SepaFlash® Silica Flash Column, Eluent of 0~10% MeOH/DCM@ 30 mL/min) to give compound **5** (220 mg, 872 μmol, 61.1% yield) as a yellow oil. LC/MS: m/z = 253.3 $(M+H)^+$.

To a solution of compound **5** (220 mg, 872 μmol, 1.00 eq), HCl/dioxane (2 M, 4.00 mL, 9.18 eq) in CH_2Cl_2 (2.00 mL) was added HCl/dioxane (2 M, 4.00 mL, 9.18 eq). The mixture was stirred at 25 °C for 12 h. The reaction mixture was concentrated under reduced pressure to give compound **11g** (200 mg, crude) as a yellow oil. LC/MS: m/z = 153.2 $(M+H)^+$.

Supporting Figures

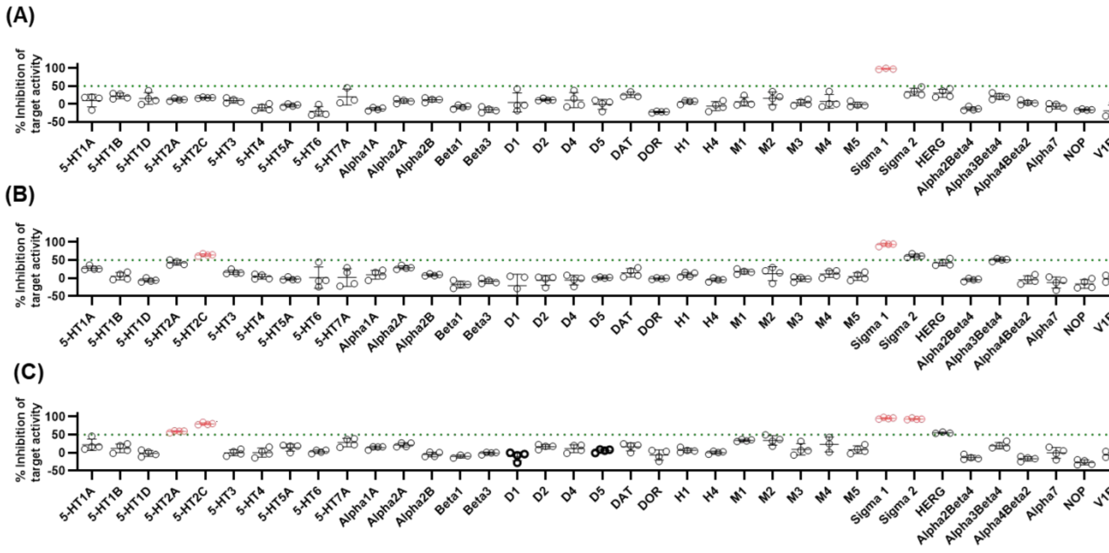

Figure S1. Off-target pharmacological evaluation of compound **2** (A), compound **3** (B), and compound **4** (C) at a concentration of 10 μM against major CNS targets, including common GPCRs, enzymes, ion channels and transporters. All data are presented as mean ± SD, n = 4.

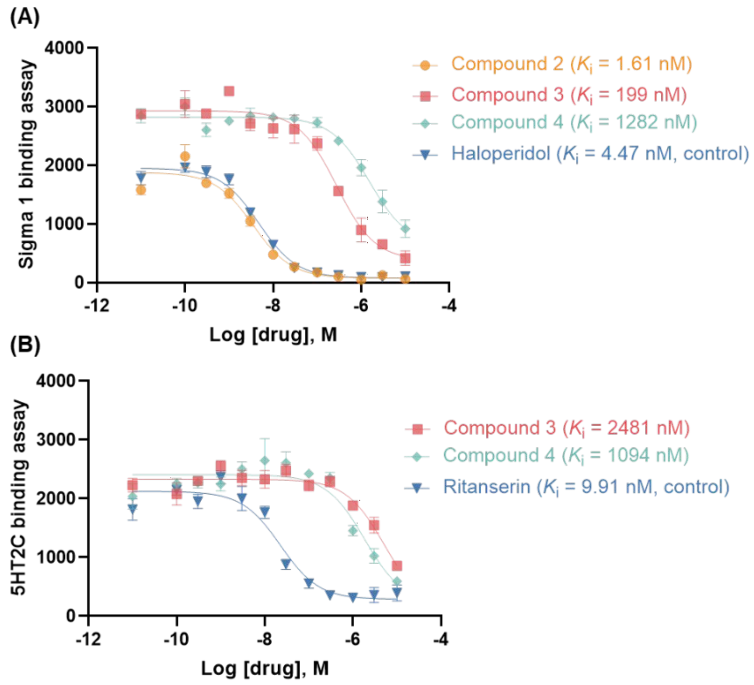

Figure S2. The binding affinities between reference compounds and CNS targets. (A) sigma 1 binding assay; (B) $5HT_{2C}$ binding assay. All data are presented as mean ± SD, n = 4.

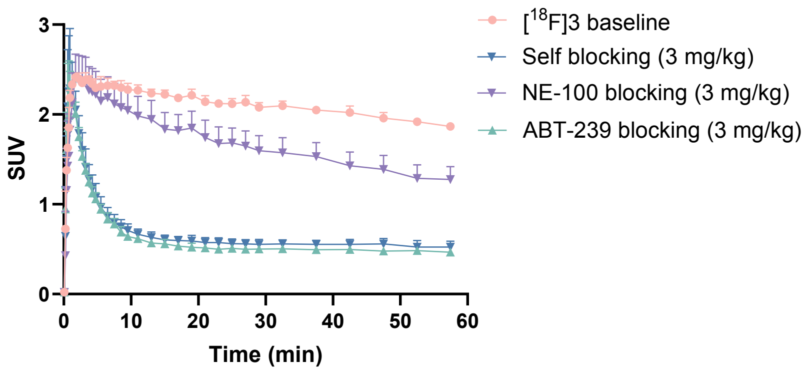

Figure S3. TACs of **[^{18}F]3** in the whole-brain under baseline blocking conditions (self-blocking, NE-100 blocking, and ABT-239 blocking: 3 mg/kg).

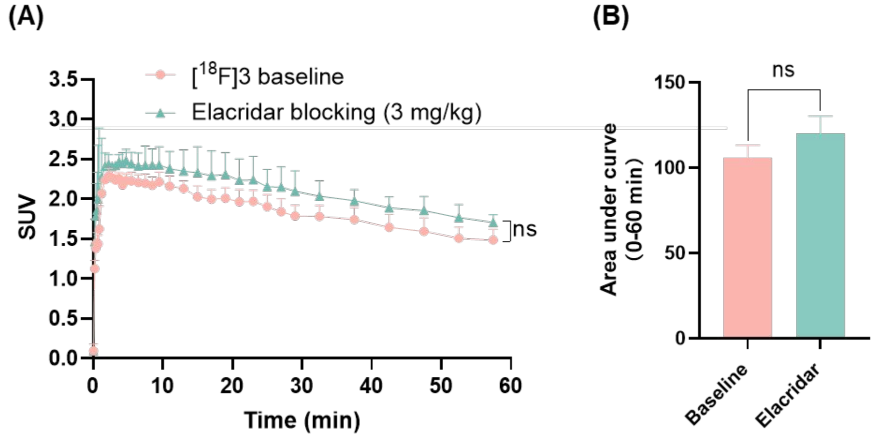

Figure S4. Mice PET imaging studies (0−60 minutes) of **[18F]3**. (A) TACs of **[18F]3** in the whole-brain under baseline and elacridar-blocking (3 mg/kg) conditions; (B) Area under curve of **[18F]3** in the whole-brain; All data were referred to as mean ± SD, n = 2, ns, not significant.

Molar activity

The molar activities of [18F]3 and [18F]4 were determined using calibration curves, which were generated by plotting the concentration of the authentic standard compounds 3 or 4 against their respective flow injection analysis peak area ratios (Figure S4). Based on these calibration curves, the molar activities of [18F]3 and [18F]4 were calculated and reported as GBq/µmol.

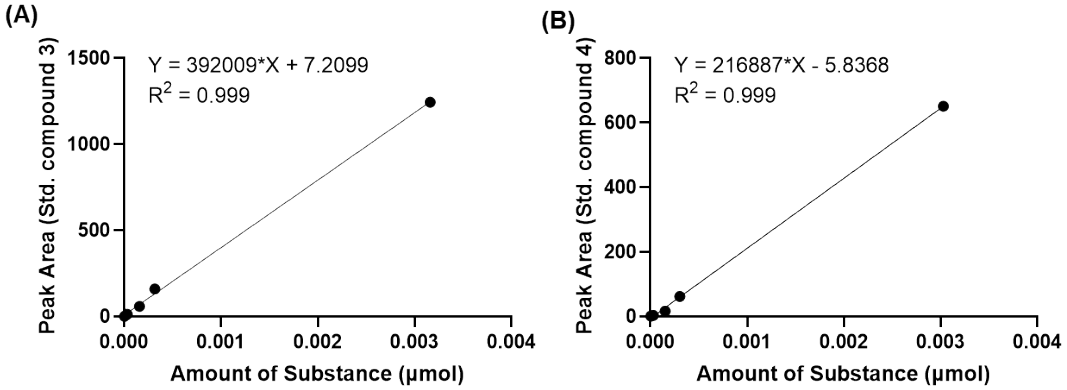

Figure S5. Calibration curves of compounds **3** (A) and **4** (B). The standard curve was generated by plotting the HPLC UV signal area against concentrations of the compounds **3** or **4**. Linear regression analysis was performed to determine the correlation between peak area and concentration.

Quantification of specific tracer binding was performed using volume of distribution (V_T) values, derived from compartmental modeling and an image-derived input function. This approach provided an estimation of V_T values and demonstrated a significant reduction in H_3R-rich brain regions such as the cortex and striatum under blocking conditions with GSK189254, enerisant, and cipralisant, confirming the specificity of [^{18}F]**3** binding (**Table S1**).

Table S1. Estimation of V_T values by Logan analysis in mice with **[^{18}F]3** at baseline, GSK189254-blocking (3 mg/kg), enerisant-blocking (3 mg/kg), and cipralisant-blocking (3 mg/kg) conditions.

Group	Baseline	GSK189254 blocking	Enerisant blocking	Cipralisant blocking
Whole brain	5.29 ± 0.52	3.40 ± 0.44	3.58 ± 0.18	2.61 ± 0.30
Cortex	4.82 ± 0.09	2.17 ± 0.08	3.18 ± 0.12	2.38 ± 0.34
Striatum	5.64 ± 0.02	2.91 ± 0.05	3.48 ± 0.27	2.67 ± 0.33
Thalamus	6.71 ± 0.05	3.90 ± 0.90	4.11 ± 0.26	2.78 ± 0.40
Hippocampus	5.80 ± 0.05	3.77 ± 0.47	3.84 ± 0.19	2.61 ± 0.32
Cerebellum	5.59 ± 0.37	3.85 ± 0.61	3.73 ± 0.16	2.94 ± 0.33

^{1}H NMR, ^{13}C NMR, and ^{19}F NMR spectra

^{1}H NMR spectra of (4-cyclopropylpiperazin-1-yl)((1S,2S)-2-(4-fluorophenyl)cyclopropyl)methanone (**1**) in $CDCl_3$.

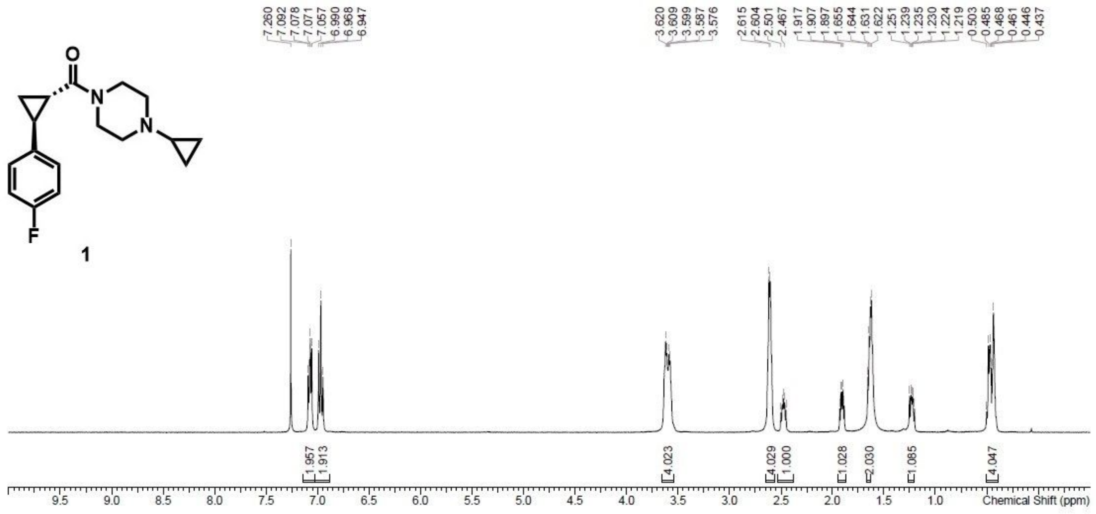

^{13}C NMR spectra of (4-cyclopropylpiperazin-1-yl)((1S,2S)-2-(4-fluorophenyl)cyclopropyl)methanone (**1**) in $CDCl_3$.

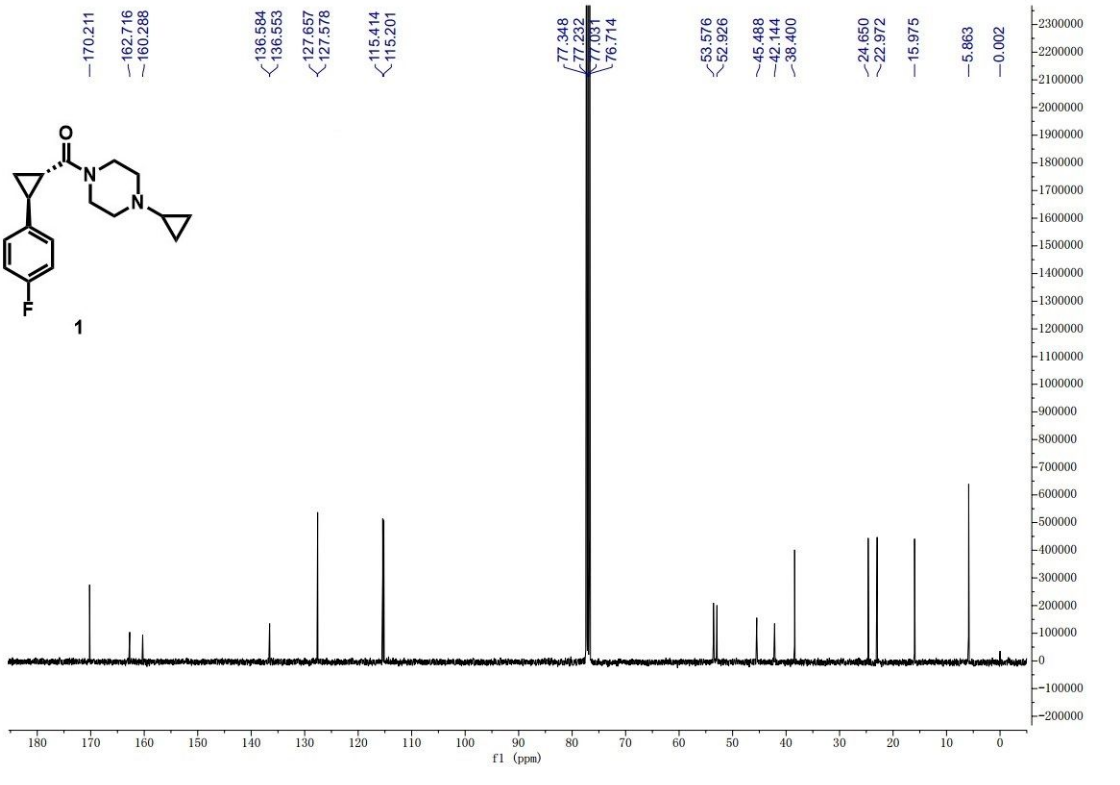

^{19}F NMR spectra of (4-cyclopropylpiperazin-1-yl)((1S,2S)-2-(4-fluorophenyl)cyclopropyl)methanone (**1**) in $CDCl_3$.

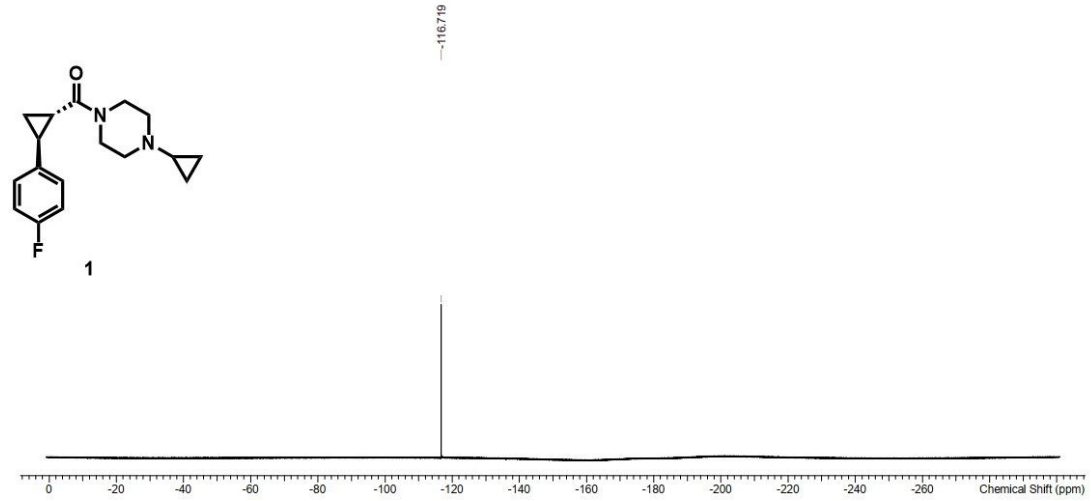

^{1}H NMR spectra of (4-cyclobutylpiperazin-1-yl)((1S,2S)-2-(4-fluorophenyl)cyclopropyl)methanone (**2**) in MeOH.

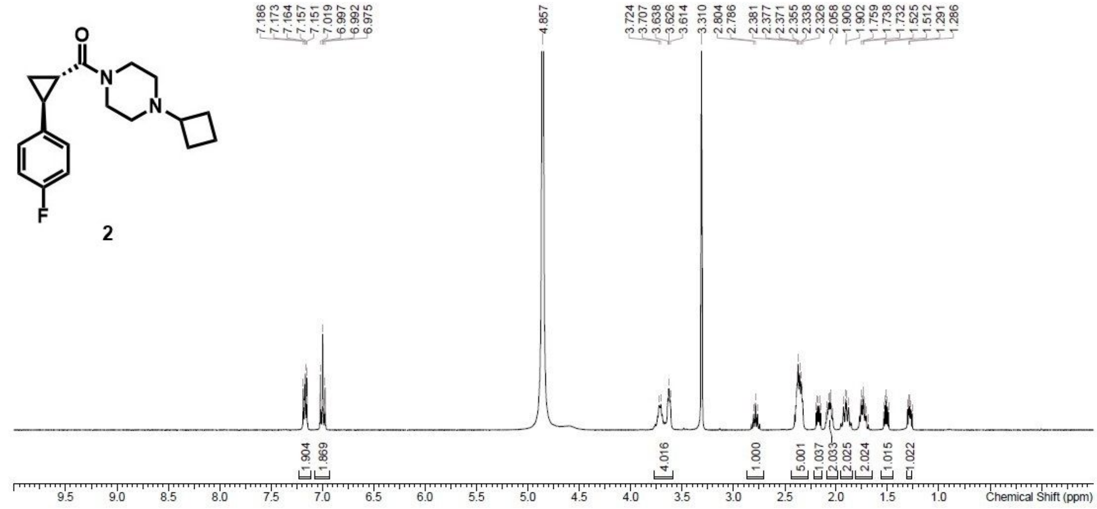

^{13}C NMR spectra of (4-cyclobutylpiperazin-1-yl)((1S,2S)-2-(4-fluorophenyl)cyclopropyl)methanone (**2**) in MeOH.

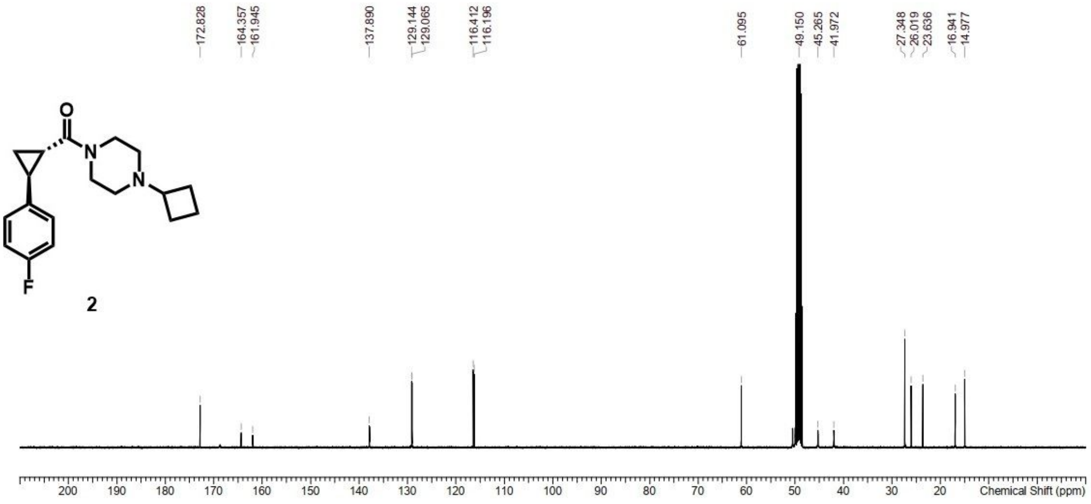

^{19}F NMR spectra of (4-cyclobutylpiperazin-1-yl)((1S,2S)-2-(4-fluorophenyl)cyclopropyl)methanone (**2**) in MeOH.

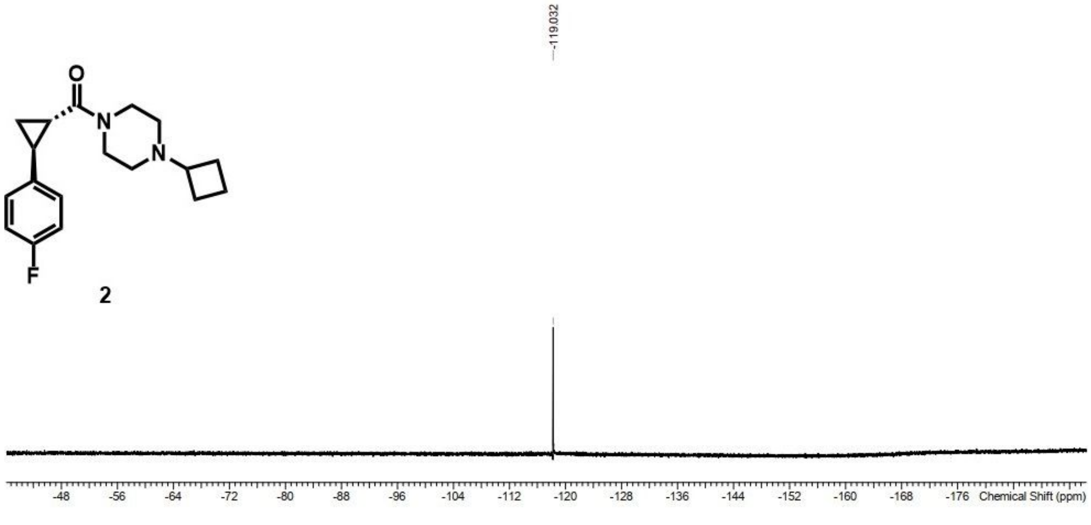

^{1}H NMR spectra of (4-cyclopentylpiperazin-1-yl)((1S,2S)-2-(4-fluorophenyl)cyclopropyl)methanone (**3**) in $CDCl_3$.

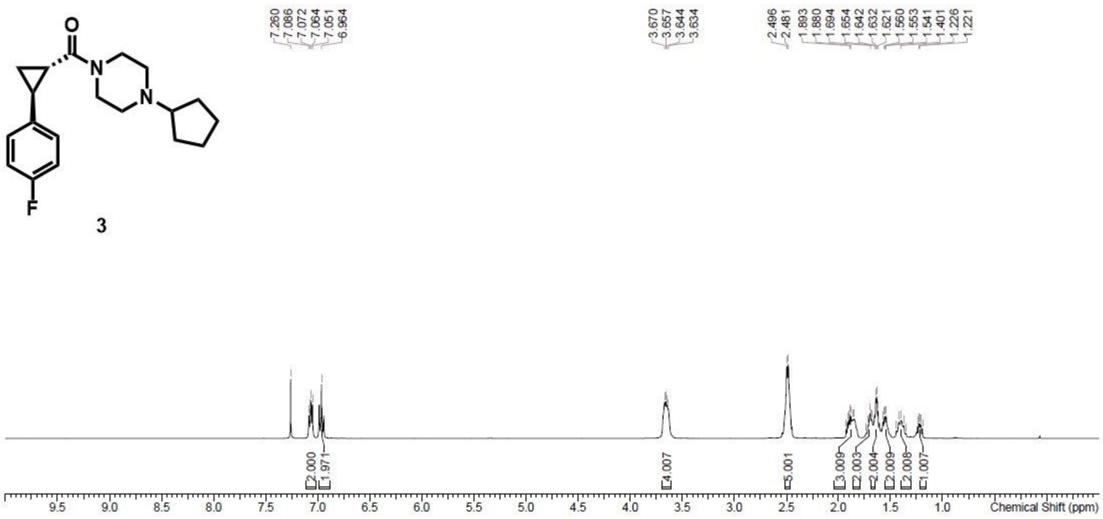

^{13}C NMR spectra of (4-cyclopentylpiperazin-1-yl)((1S,2S)-2-(4-fluorophenyl)cyclopropyl)methanone (**3**) in $CDCl_3$.

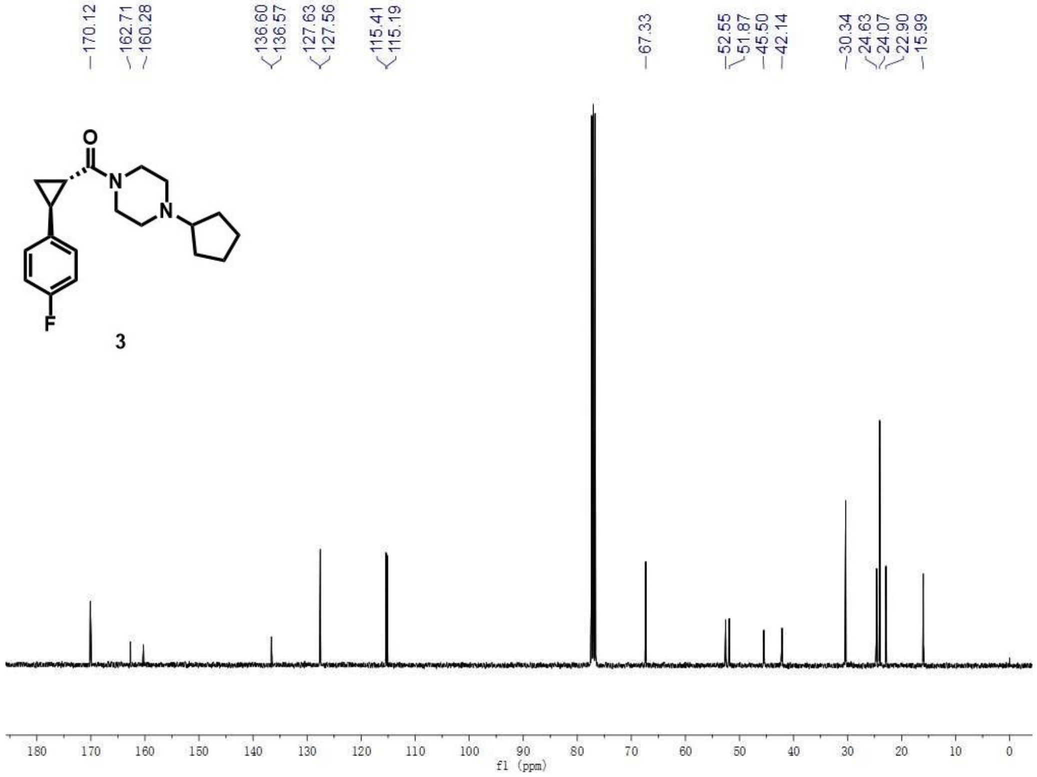

^{19}F NMR spectra of (4-cyclopentylpiperazin-1-yl)((1S,2S)-2-(4-fluorophenyl)cyclopropyl)methanone (**3**) in $CDCl_3$.

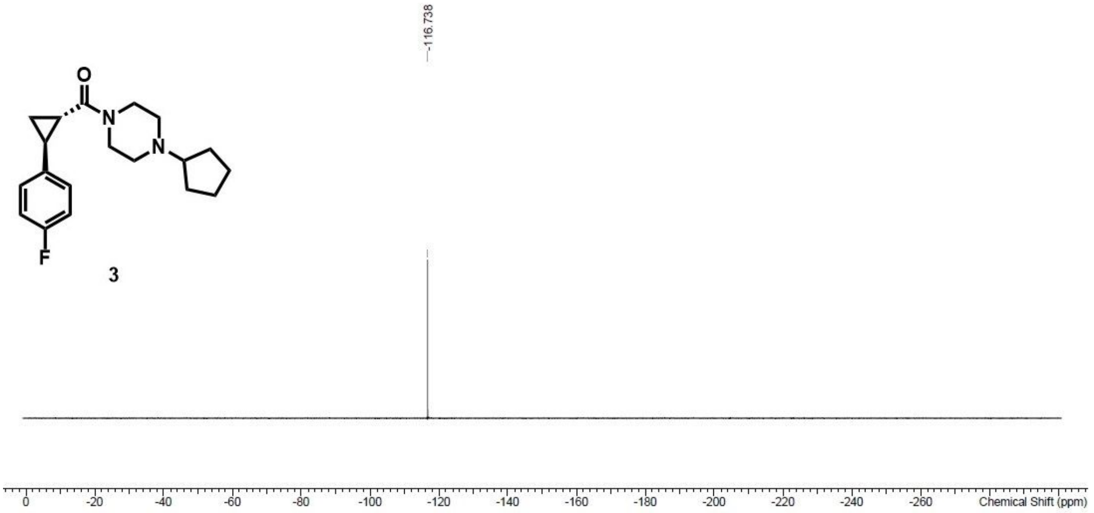

^{1}H NMR spectra of (4-cyclohexylpiperazin-1-yl)((1S,2S)-2-(4-fluorophenyl)cyclopropyl)methanone (**4**) in $CDCl_3$.

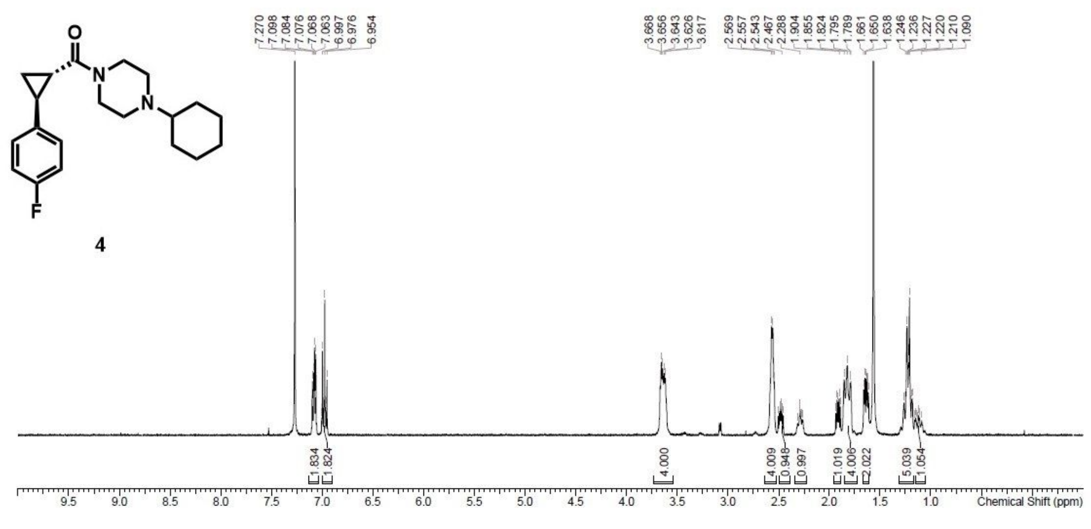

^{13}C NMR spectra of (4-cyclohexylpiperazin-1-yl)((1S,2S)-2-(4-fluorophenyl)cyclopropyl)methanone (**4**) in $CDCl_3$.

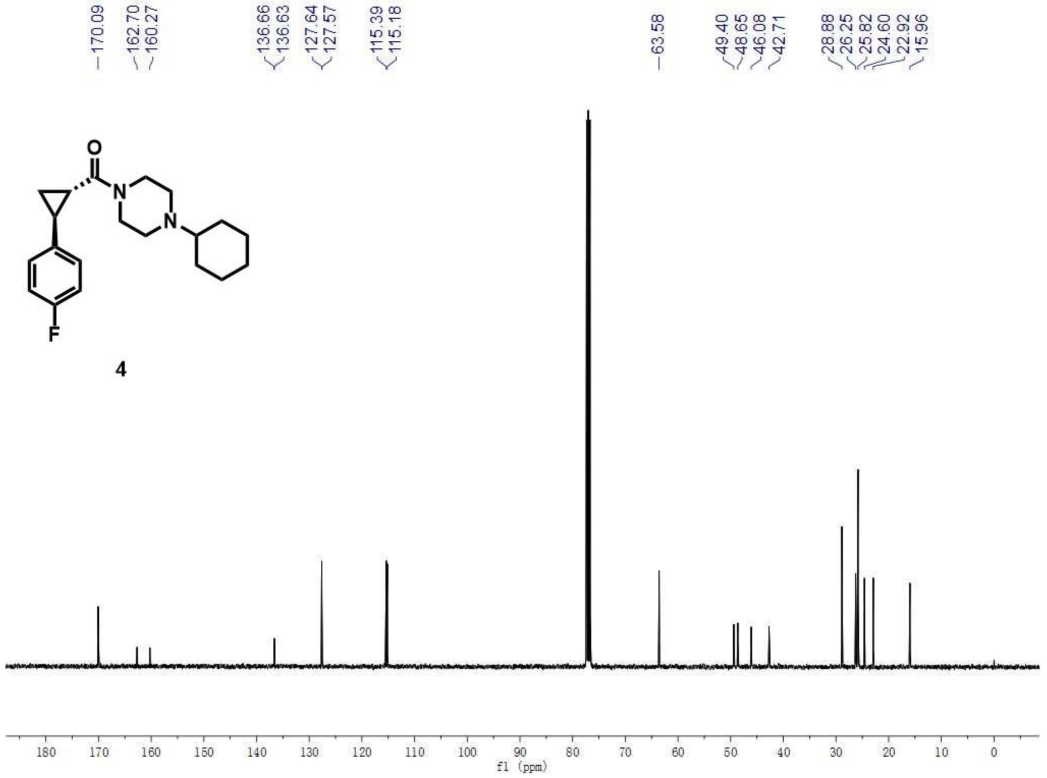

^{13}F NMR spectra of (4-cyclohexylpiperazin-1-yl)((1S,2S)-2-(4-fluorophenyl)cyclopropyl)methanone (**4**) in $CDCl_3$.

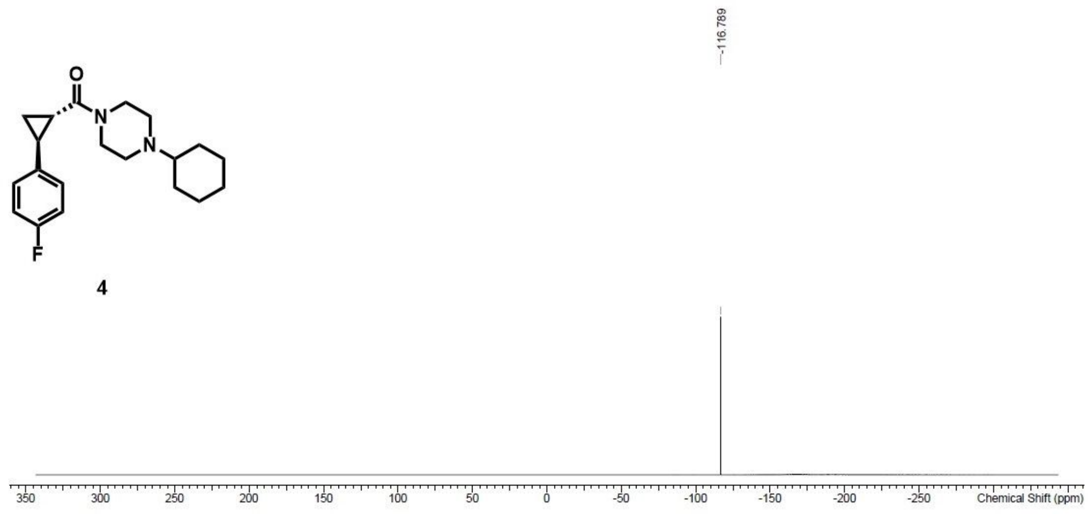

^{1}H NMR spectra of (4-cycloheptylpiperazin-1-yl)((1S,2S)-2-(4-fluorophenyl)cyclopropyl)methanone (**5**) in $CDCl_3$.

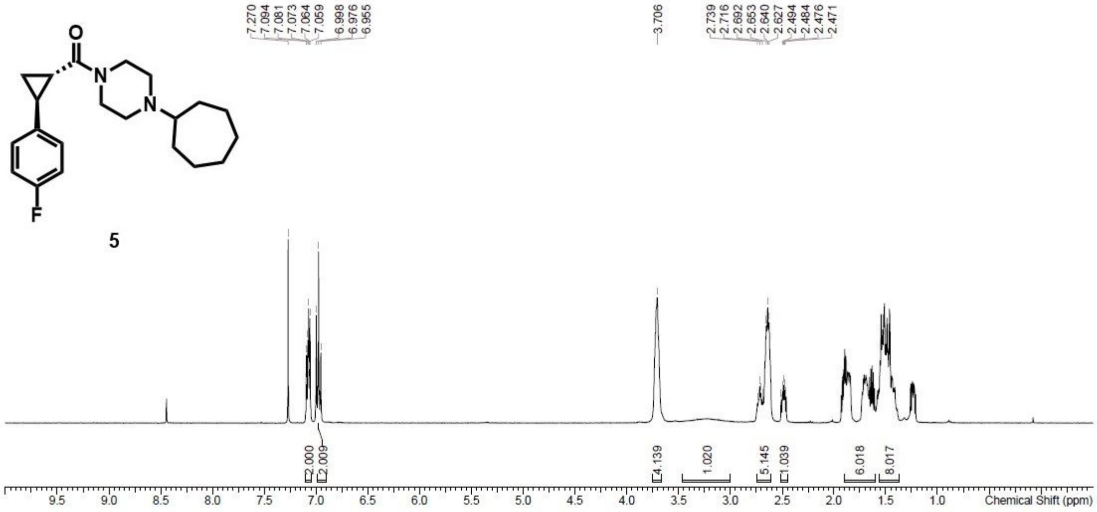

^{13}C NMR spectra of (4-cycloheptylpiperazin-1-yl)((1S,2S)-2-(4-fluorophenyl)cyclopropyl)methanone (**5**) in $CDCl_3$.

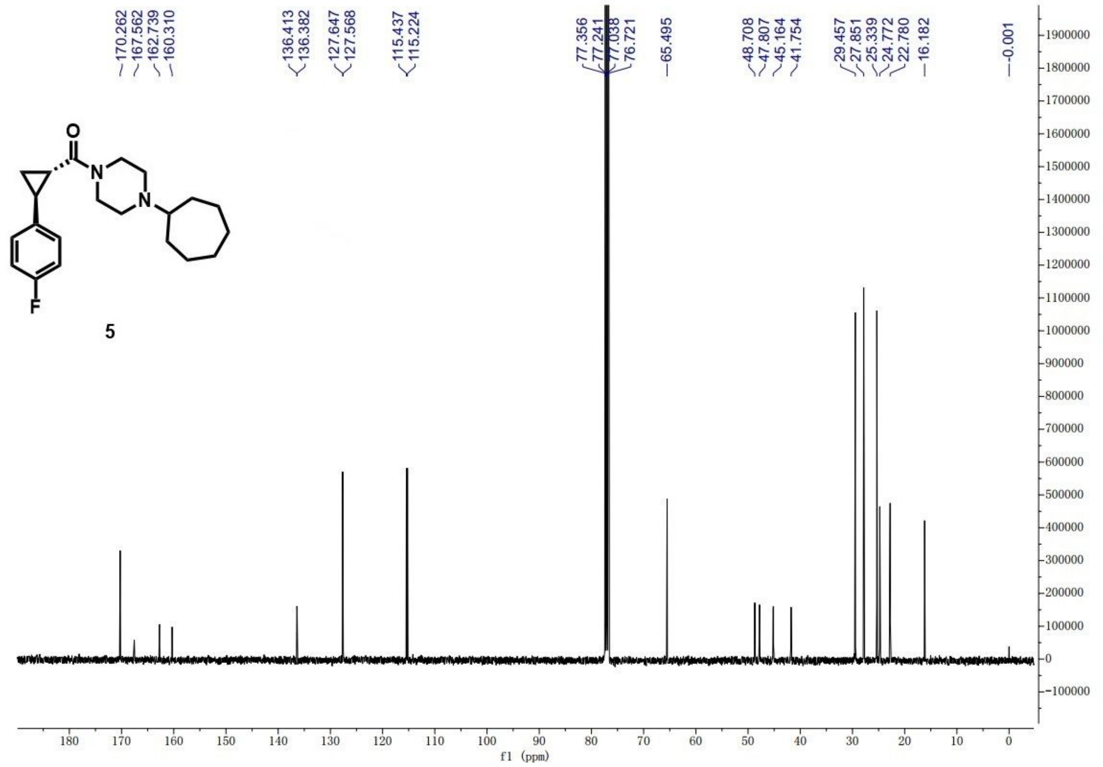

^{19}F NMR spectra of (4-cycloheptylpiperazin-1-yl)((1S,2S)-2-(4-fluorophenyl)cyclopropyl)methanone (**5**) in $CDCl_3$.

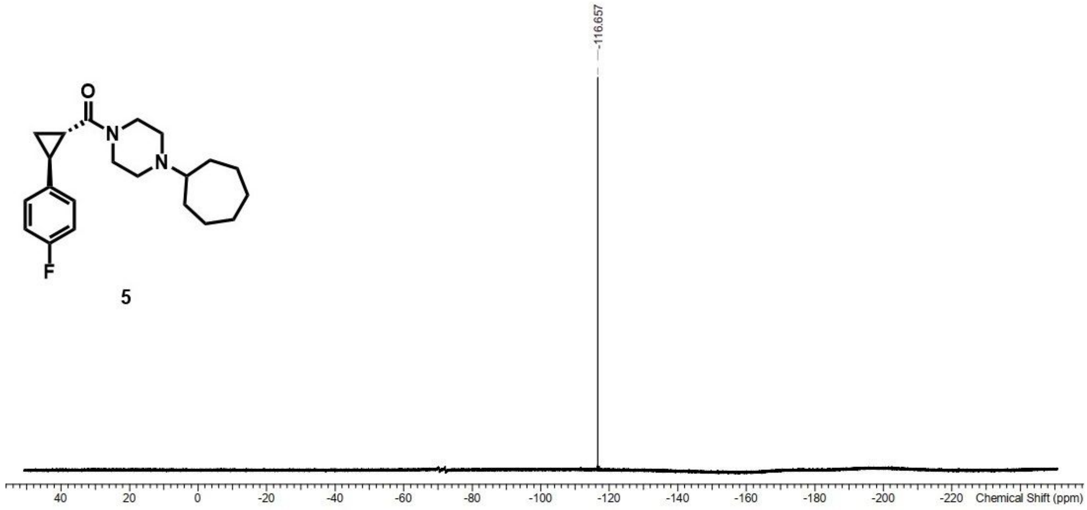

^{1}H NMR spectra of (6-cyclobutyl-2,6-diazaspiro[3.3]heptan-2-yl)((1S,2S)-2-(4-fluorophenyl)cyclopropyl)methanone (**6**) in $CDCl_3$.

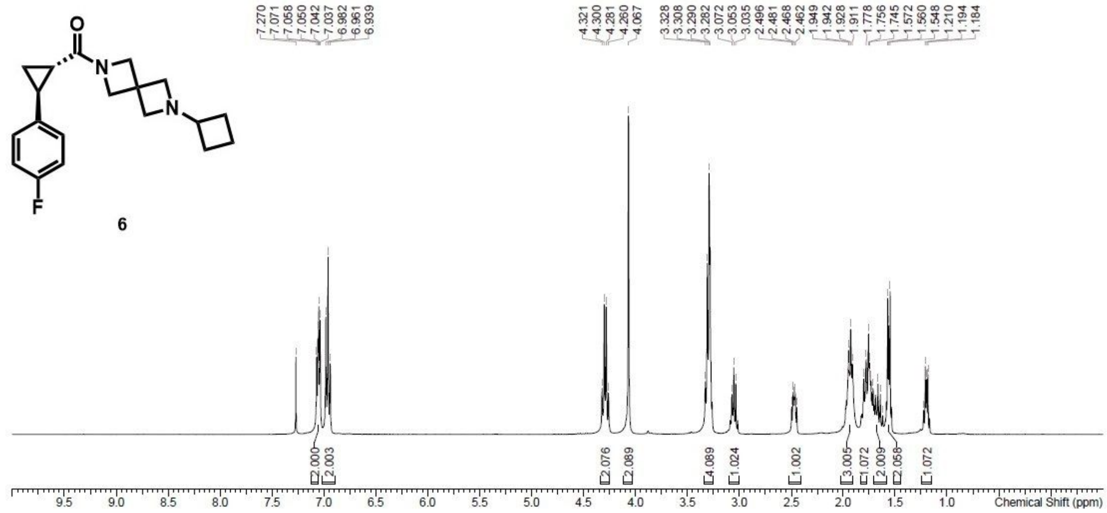

^{13}C NMR spectra of (6-cyclobutyl-2,6-diazaspiro[3.3]heptan-2-yl)((1S,2S)-2-(4-fluorophenyl)cyclopropyl)methanone (**6**) in $CDCl_3$.

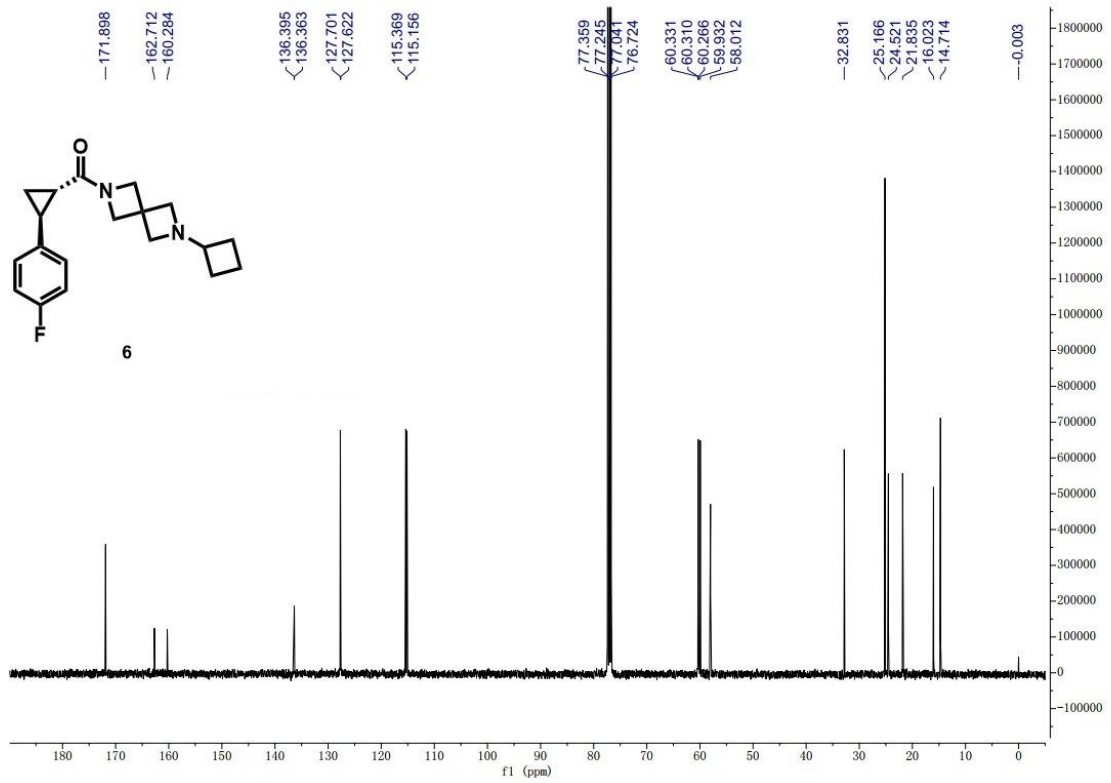

^{19}F NMR spectra of (6-cyclobutyl-2,6-diazaspiro[3.3]heptan-2-yl)((1S,2S)-2-(4-fluorophenyl)cyclopropyl)methanone (**6**) in $CDCl_3$.

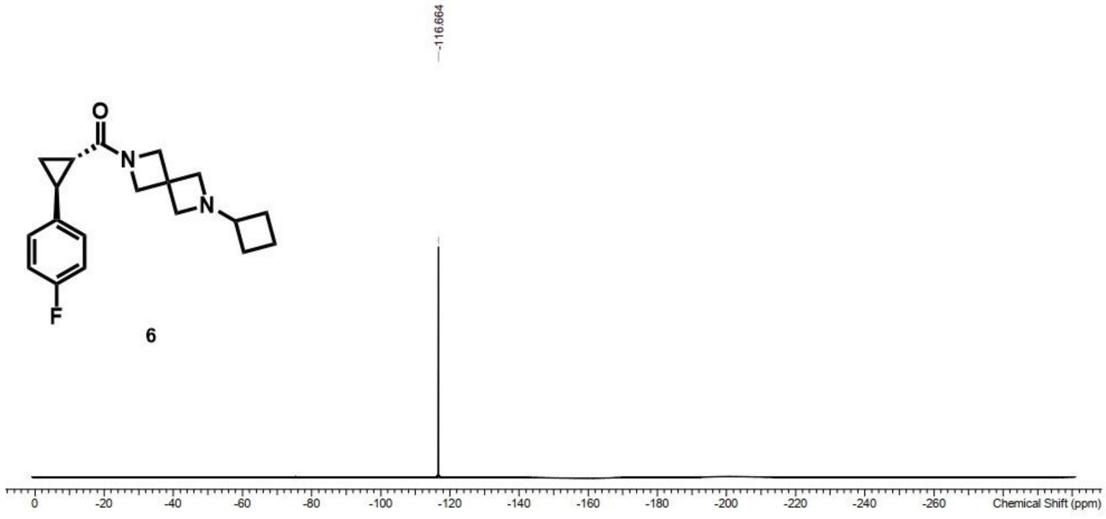

^{1}H NMR spectra of ((1S,4S)-5-cyclobutyl-2,5-diazabicyclo[2.2.1]heptan-2-yl)((1S,2S)-2-(4-fluorophenyl)cyclopropyl)methanone (**7**) in $CDCl_3$.

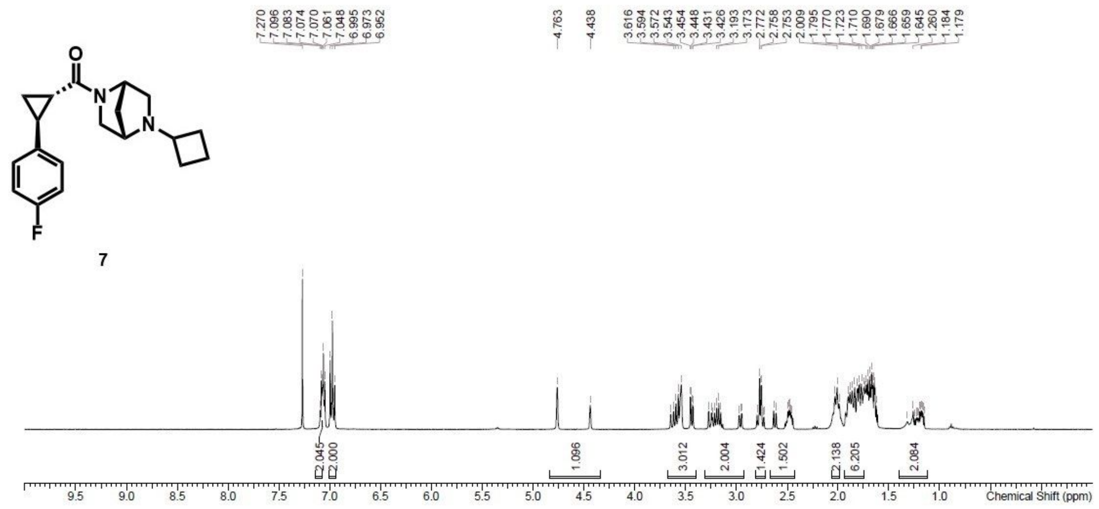

^{19}F NMR spectra of ((1S,4S)-5-cyclobutyl-2,5-diazabicyclo[2.2.1]heptan-2-yl)((1S,2S)-2-(4-fluorophenyl)cyclopropyl)methanone (**7**) in $CDCl_3$.

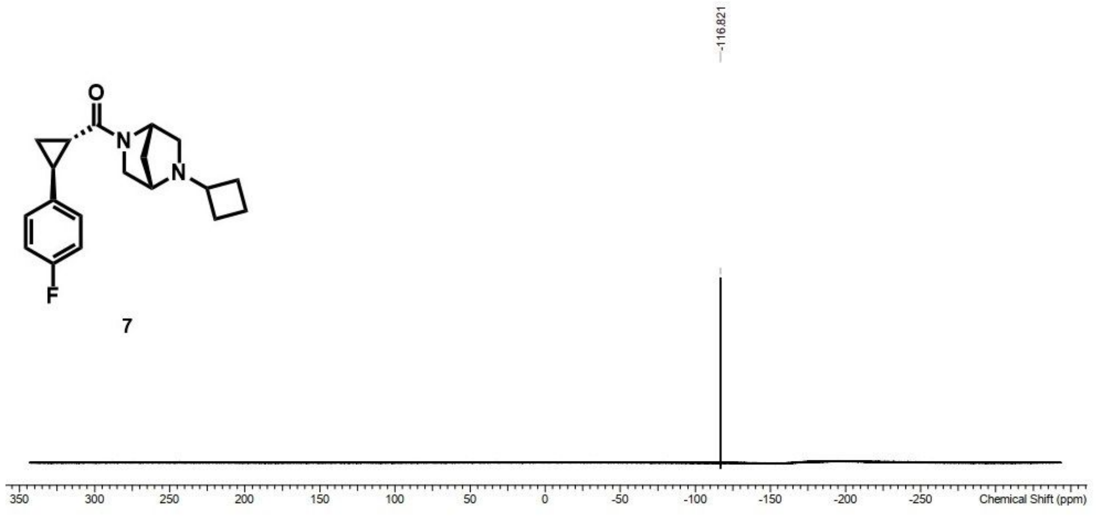

^{1}H NMR spectra of ((3aR,6aS)-5-cyclobutylhexahydropyrrolo[3,4-c]pyrrol-2(1H)-yl)((1S,2S)-2-(4-fluorophenyl)cyclopropyl)methanone (**8**) in $CDCl_3$.

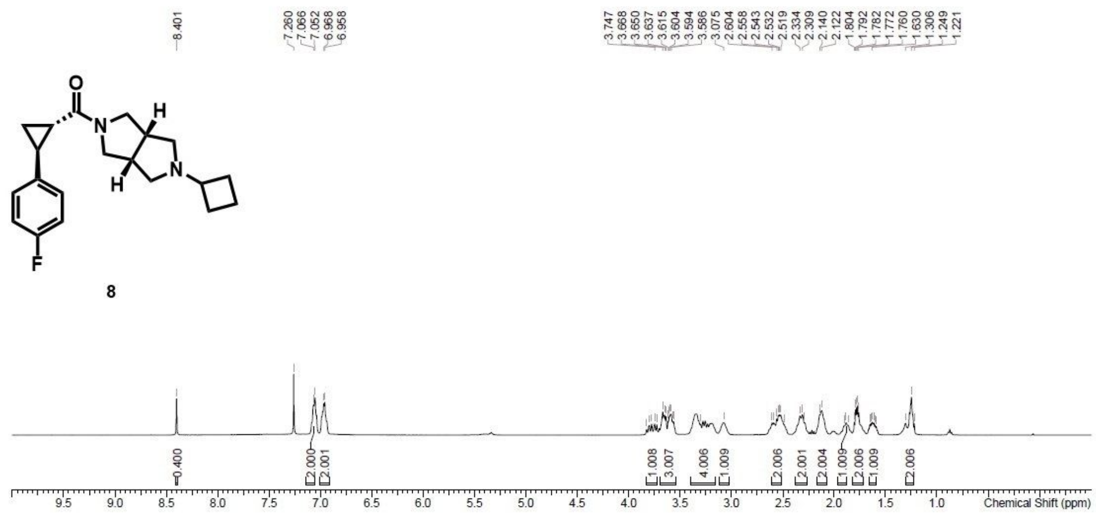

^{19}F NMR spectra of ((3aR,6aS)-5-cyclobutylhexahydropyrrolo[3,4-c]pyrrol-2(1H)-yl)((1S,2S)-2-(4-fluorophenyl)cyclopropyl)methanone (**8**) in $CDCl_3$.

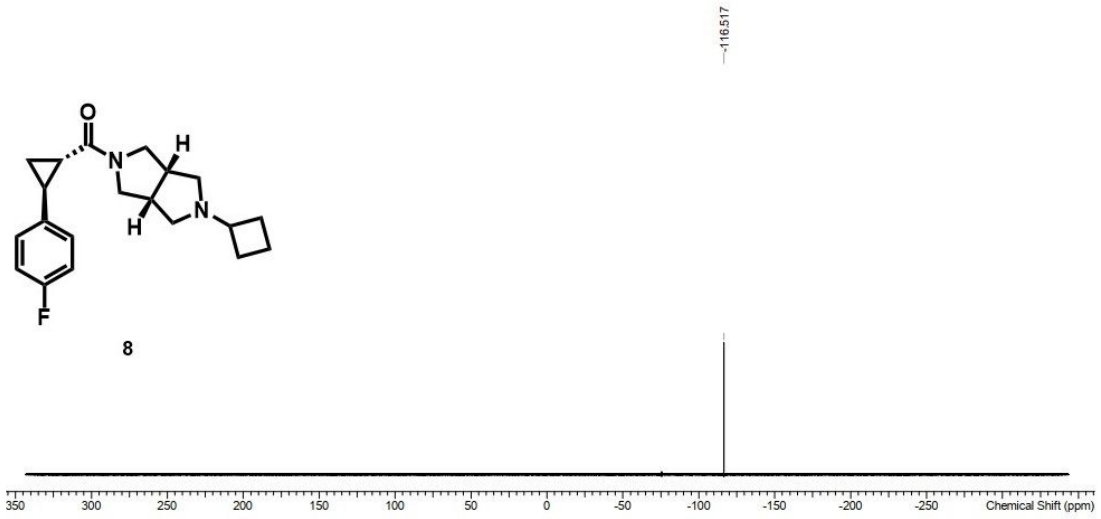

^{1}H NMR spectra of (4-cyclobutylpiperazin-1-yl)((1S,2S)-2-(4-(3-fluoroazetidine-1-carbonyl)phenyl)cyclopropyl)methanone (**9**) in $CDCl_3$.

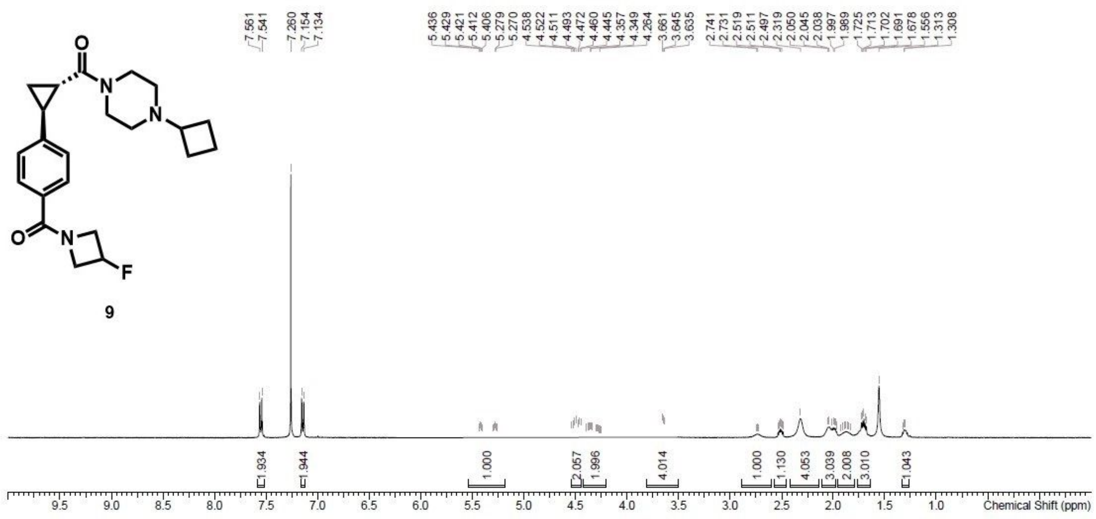

^{13}C NMR spectra of (4-cyclobutylpiperazin-1-yl)((1S,2S)-2-(4-(3-fluoroazetidine-1-carbonyl)phenyl)cyclopropyl)methanone (**9**) in $CDCl_3$.

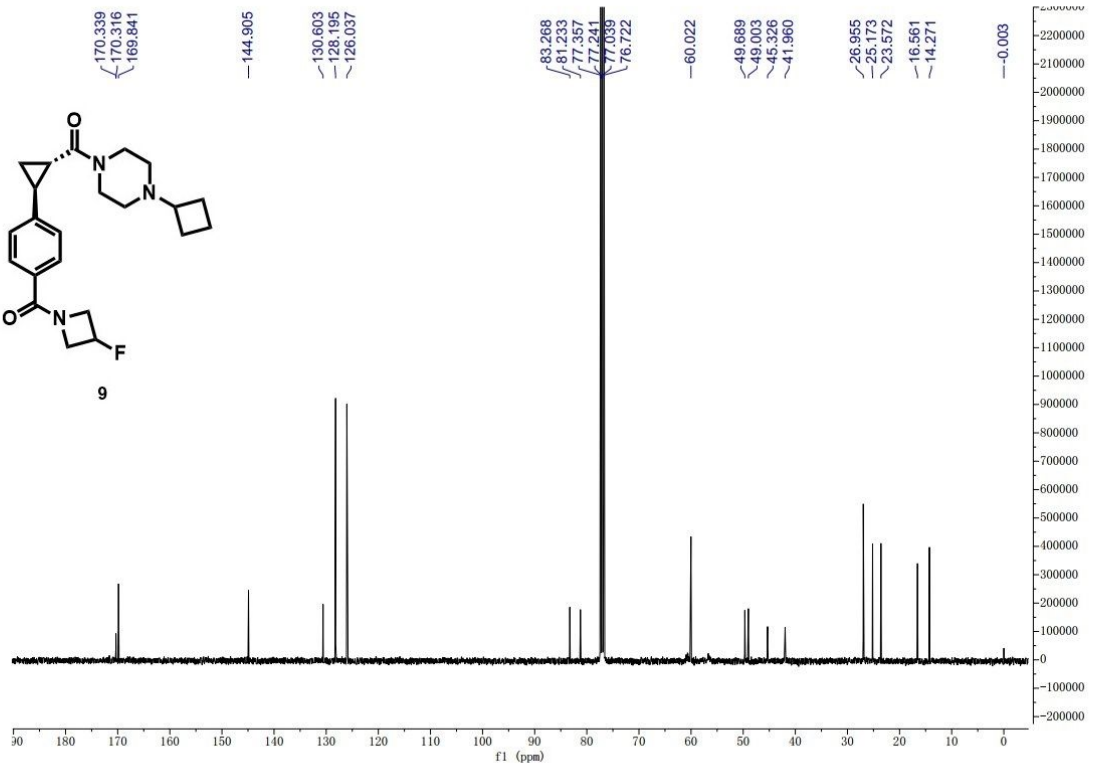

^{19}F NMR spectra of (4-cyclobutylpiperazin-1-yl)((1S,2S)-2-(4-(3-fluoroazetidine-1-carbonyl)phenyl)cyclopropyl)methanone (**9**) in $CDCl_3$.

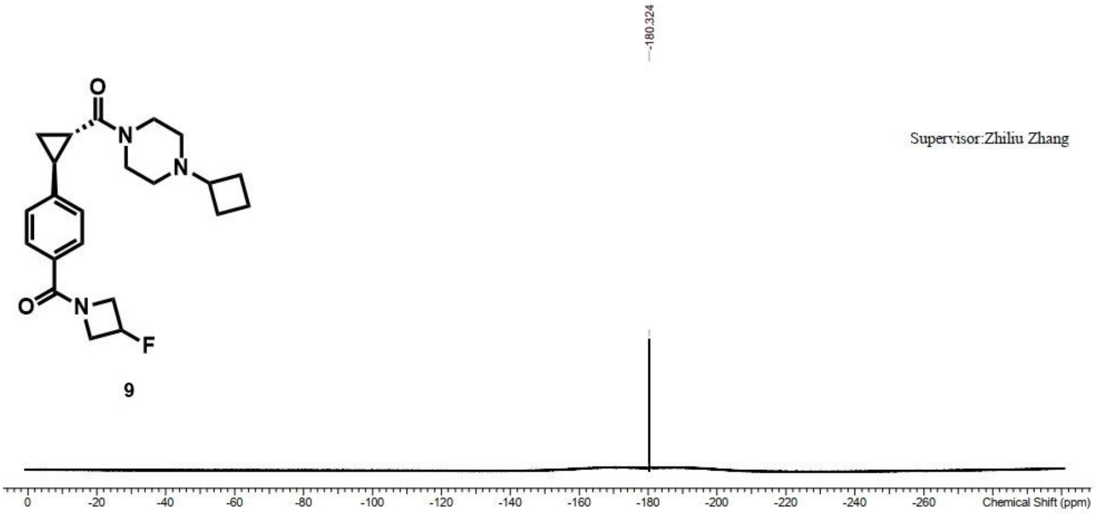

^{1}H NMR spectra of (4-cyclopentylpiperazin-1-yl)((1S,2S)-2-(4-(4,4,5,5-tetramethyl-1,3,2-dioxaborolan-2-yl)phenyl)cyclopropyl)methanone (**22**) in $CDCl_3$.

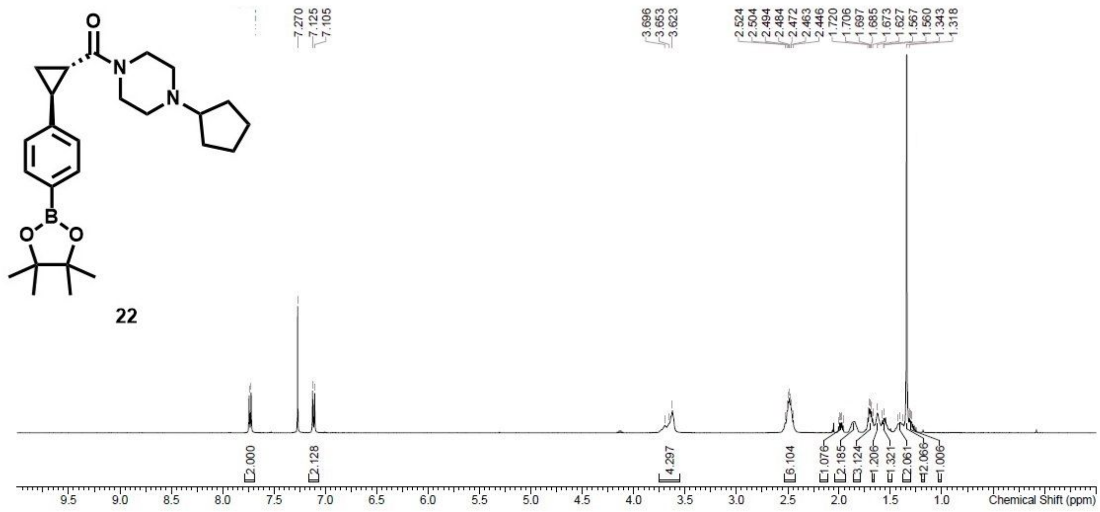

^{13}C NMR spectra of (4-cyclopentylpiperazin-1-yl)((1S,2S)-2-(4-(4,4,5,5-tetramethyl-1,3,2-dioxaborolan-2-yl)phenyl)cyclopropyl)methanone (**22**) in $CDCl_3$.

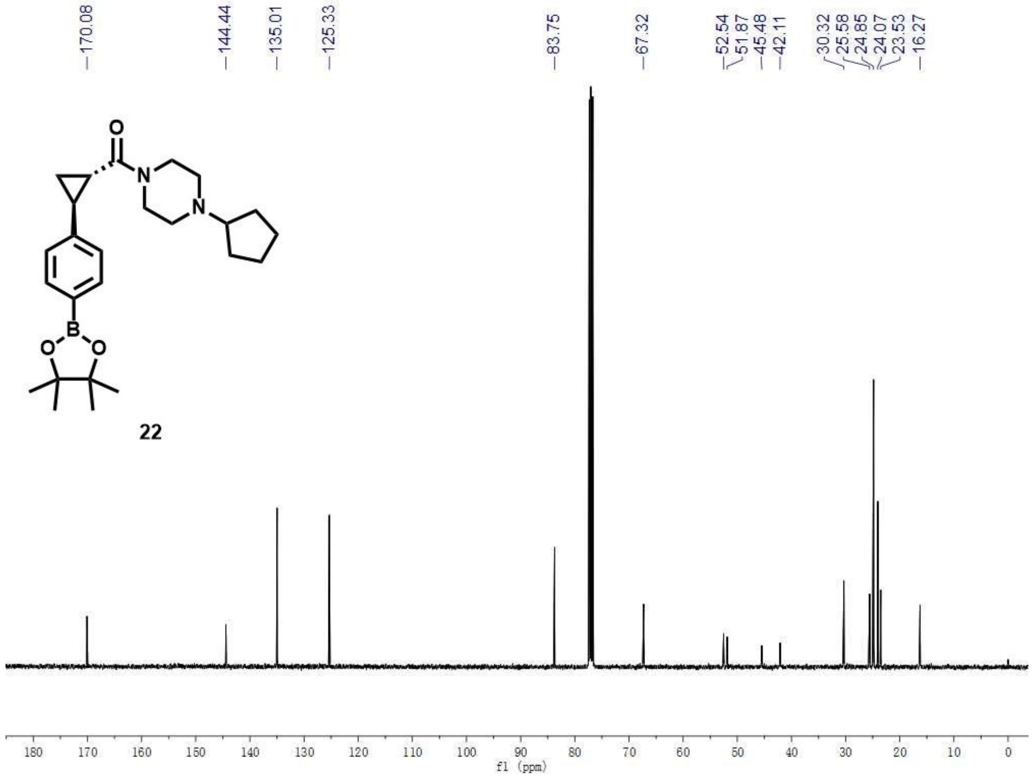

^{1}H NMR spectra of (4-cyclohexylpiperazin-1-yl)((1S,2S)-2-(4-(4,4,5,5-tetramethyl-1,3,2-dioxaborolan-2-yl)phenyl)cyclopropyl)methanone (**24**) in $CDCl_3$.

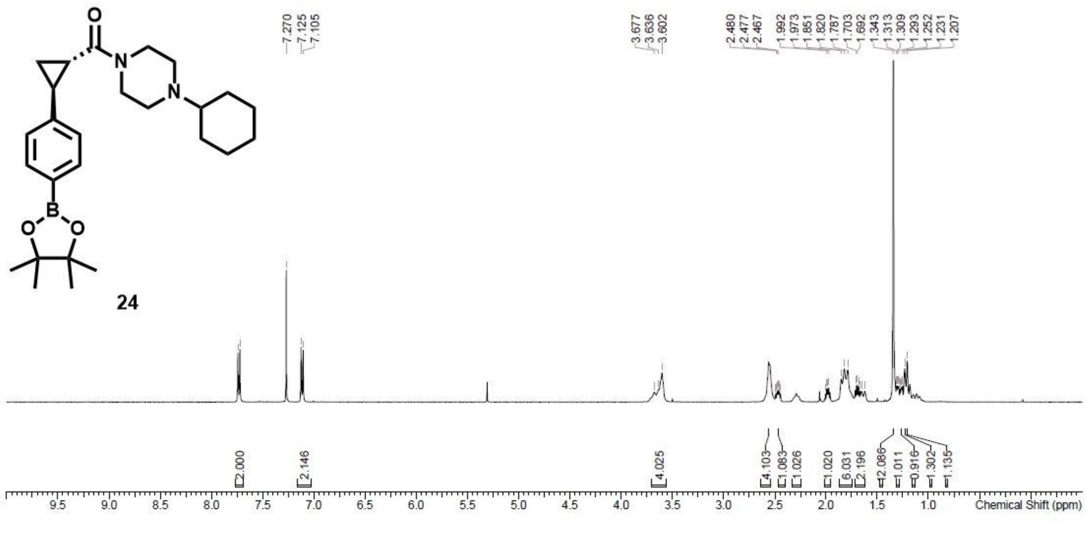

^{13}C NMR spectra of (4-cyclohexylpiperazin-1-yl)((1S,2S)-2-(4-(4,4,5,5-tetramethyl-1,3,2-dioxaborolan-2-yl)phenyl)cyclopropyl)methanone (**24**) in $CDCl_3$.

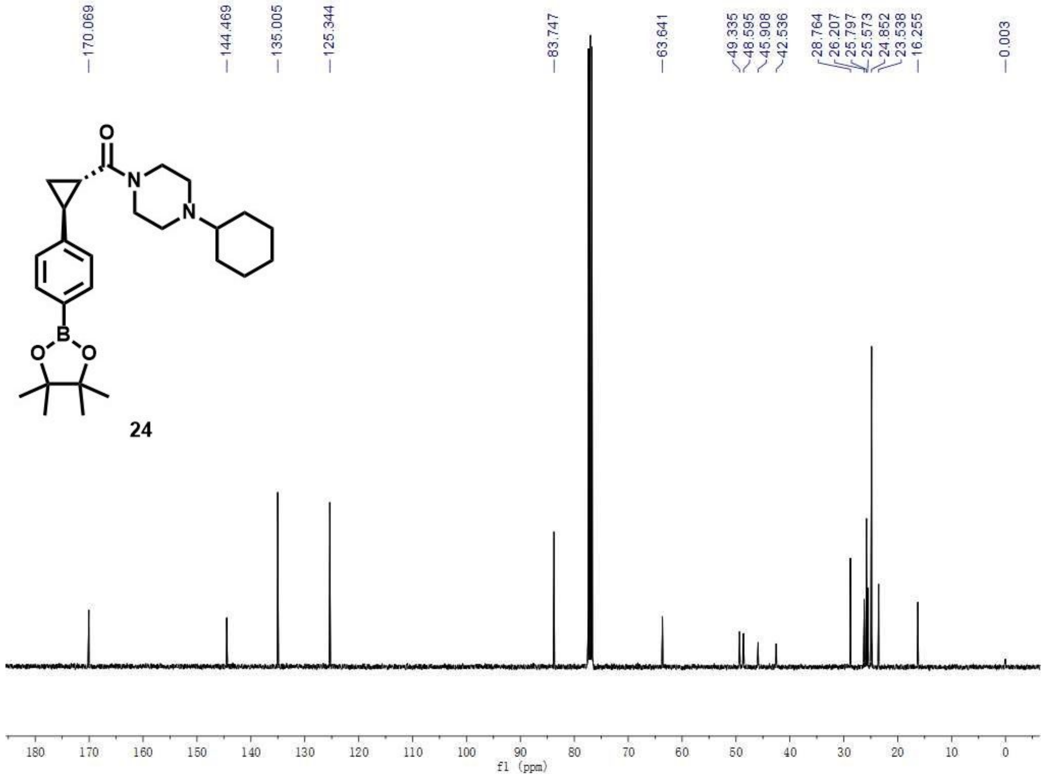

HPLC analysis of Compounds 3 and 4

Compounds 3 and 4 are > 95% purity confirmed by Shimadzu LC-20AD HPLC [mobile phase: ramp from 3% ACN (0.02% TFA) in water (0.04% TFA) to 60% ACN in water in 8.0 min; again from 60% ACN (0.02% TFA) in water (0.04% TFA) to 80% ACN in water in 2.0 min; return back to 3% ACN (0.02%TFA) and hold for 3.0 minutes. Flow rate: 0.5mL/min, column temperature: 40 ℃, and detector wavelength at 220 nm, 254 nm and 215 nm. Column: Halo C18 3.0 × 100 mm, 2.7 μm].

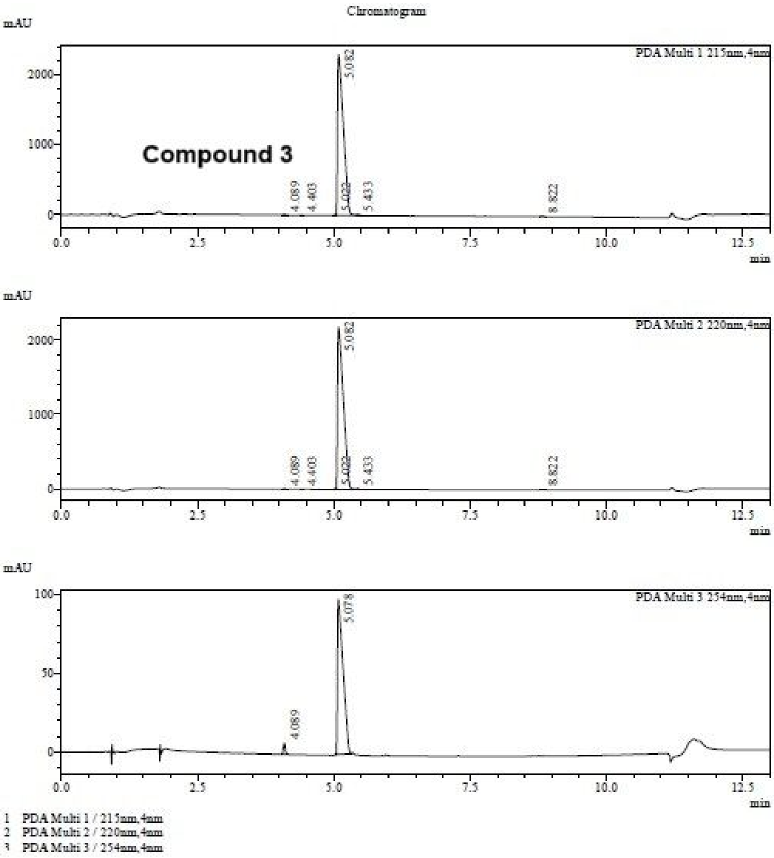

==

Peak Table

PDA Ch1 215nm

Peak#	Ret. Time	Height	Height%	USP Width	Area	Area%
1	4.089	10052	0.432	0.063	22923	0.131
2	4.403	2133	0.092	0.054	3977	0.023
3	5.022	12717	0.546	0.046	18299	0.105
4	5.082	2285749	98.138	0.223	17394014	99.540
5	5.433	11930	0.512	0.051	20744	0.119
6	8.822	6527	0.280	0.062	14404	0.082

PDA Ch2 220nm

Peak#	Ret. Time	Height	Height%	USP Width	Area	Area%
1	4.089	9158	0.414	0.063	20680	0.125
2	4.403	2016	0.091	0.054	3735	0.023
3	5.022	12428	0.562	0.046	17944	0.108
4	5.082	2171656	98.136	0.223	16499855	99.541
5	5.433	11342	0.513	0.051	19719	0.119
6	8.822	6305	0.285	0.062	13929	0.084

PDA Ch3 254nm

Peak#	Ret. Time	Height	Height%	USP Width	Area	Area%
1	4.089	6964	6.622	0.063	15583	2.130
2	5.078	98211	93.378	0.220	715929	97.870

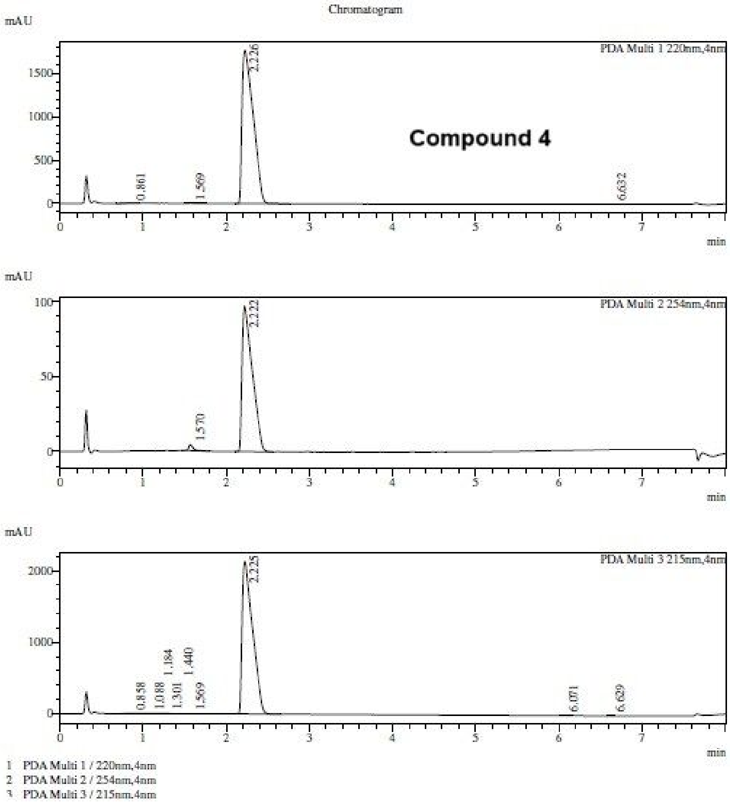

Peak Table

PDA Ch1 220nm

Peak#	Ret. Time	Height	Height%	USP Width	Area	Area%
1	0.861	1996	0.113	0.181	12945	0.078
2	1.569	1504	0.085	0.105	5512	0.033
3	2.226	1765185	99.744	0.281	16588630	99.873
4	6.632	1028	0.058	0.078	2655	0.016

PDA Ch2 254nm

Peak#	Ret. Time	Height	Height%	USP Width	Area	Area%
1	1.570	4100	4.042	0.111	16688	1.935
2	2.222	97353	95.958	0.260	845883	98.065

PDA Ch3 215nm

Peak#	Ret. Time	Height	Height%	USP Width	Area	Area%
1	0.858	5403	0.251	0.351	56839	0.298
2	1.088	2898	0.134	1.291	27554	0.144
3	1.184	2808	0.130	0.472	8346	0.044
4	1.301	2485	0.115	1.086	24970	0.131
5	1.440	1978	0.092	1.089	14222	0.075
6	1.569	2229	0.103	0.205	15408	0.081
7	2.225	2134288	99.023	0.261	18920903	99.184
8	6.071	1464	0.068	0.082	3582	0.019
9	6.629	1800	0.084	0.082	4809	0.025

Semi-prep HPLC of Radioligands [^{18}F]3 and [^{18}F]4

[^{18}F]3 was purified by Lablogic HPLC, Phenomenex Luna C-18 column [250 × 10 mm, 5 μm]. Mobile phase: 35% CH_3CN/0.1% TEA in water, flow :5 mL/min, retention time: 22 min. First panel: F-18 radioactivity counts; Second panel: UV 210 nm.

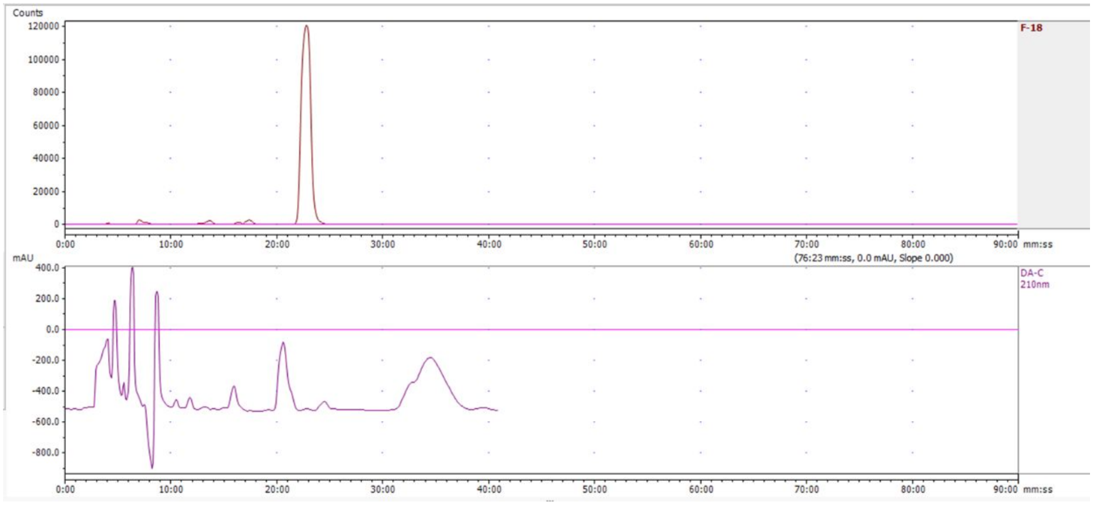

[^{18}F]4 was purified by Lablogic HPLC, Phenomenex Luna C-18 column [250 × 10 mm, 5 μm]. Mobile phase: 37% CH_3CN/0.1% TEA in water, flow :5 mL/min, retention time: 50 min. First panel: F-18 radioactivity counts; Second panel: UV 210 nm.

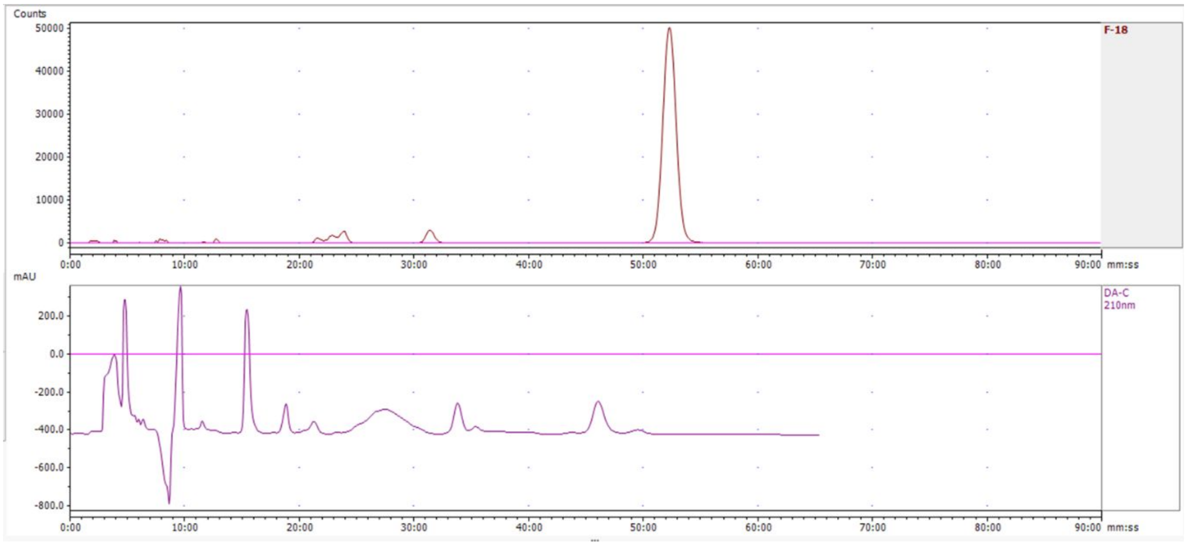

Analytical-HPLC of Radioligands [^{18}F]3 and [^{18}F]4

[^{18}F]3 was confirmed by co-injection of compound **3** via analytical HPLC (Agilent 1100 series HPLC system on Phenomenex Luna C-18 column (4.6 × 150 mm), mobile phase 25% CH_3CN/0.1% TFA in water, flow:1 mL/min). Retention time: 9.3 min. First panel: F-18 radioactivity counts; Second panel: UV 210 nm.

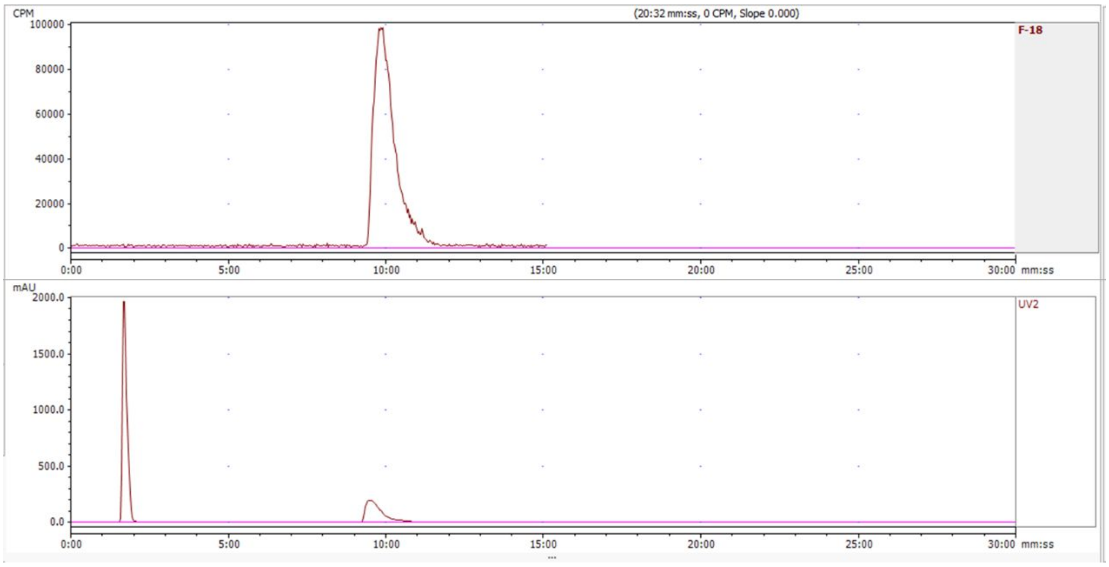

[^{18}F]4 was confirmed by co-injection of compound **4** via analytical HPLC (Agilent 1100 series HPLC system on Phenomenex Luna C-18 column (4.6 × 150 mm), mobile phase 40% CH_3CN/0.1% TFA in water, flow:1 mL/min). Retention time: 3.5 min. First panel: F-18 radioactivity counts; Second panel: UV 210 nm.

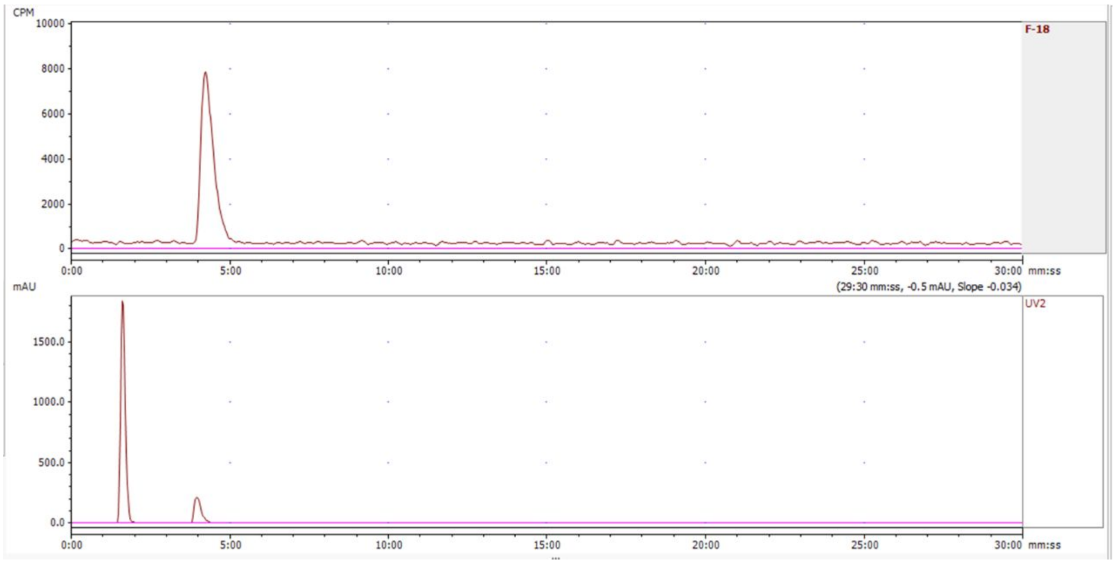

Supplement: Supplementary file 1 [file jm4c02924_si_001.pdf]
